# Neural underpinnings of preferential pain learning and the modulatory role of fear

**DOI:** 10.1093/cercor/bhad236

**Published:** 2023-07-05

**Authors:** Katarina Forkmann, Katja Wiech, Katharina Schmidt, Julia Schmid-Köhler, Ulrike Bingel

**Affiliations:** Department of Neurology, Center for Translational Neuro- and Behavioural Sciences, University Hospital Essen, University Duisburg Essen, Hufelandstraße 55, Essen 45147, Germany; Nuffield Department of Clinical Neurosciences, University of Oxford, John Radcliffe Hospital, Headley Way, Oxford OX3 9DU, United Kingdom; Department of Neurology, Center for Translational Neuro- and Behavioural Sciences, University Hospital Essen, University Duisburg Essen, Hufelandstraße 55, Essen 45147, Germany; Department of Neurology, Center for Translational Neuro- and Behavioural Sciences, University Hospital Essen, University Duisburg Essen, Hufelandstraße 55, Essen 45147, Germany; Department of Neurology, Center for Translational Neuro- and Behavioural Sciences, University Hospital Essen, University Duisburg Essen, Hufelandstraße 55, Essen 45147, Germany

**Keywords:** pain-related learning, acquisition learning, extinction learning, fear of pain, fMRI

## Abstract

Due to its unique biological relevance, pain-related learning might differ from learning from other aversive experiences. This functional magnetic resonance imaging study compared neural mechanisms underlying the acquisition and extinction of different threats in healthy humans. We investigated whether cue-pain associations are acquired faster and extinguished slower than cue associations with an equally unpleasant tone. Additionally, we studied the modulatory role of stimulus-related fear. Therefore, we used a differential conditioning paradigm, in which somatic heat pain stimuli and unpleasantness-matched auditory stimuli served as US. Our results show stronger acquisition learning for pain- than tone-predicting cues, which was augmented in participants with relatively higher levels of fear of pain. These behavioral findings were paralleled by activation of brain regions implicated in threat processing (insula, amygdala) and personal significance (ventromedial prefrontal cortex). By contrast, extinction learning seemed to be less dependent on the threat value of the US, both on the behavioral and neural levels. Amygdala activity, however, scaled with pain-related fear during extinction learning. Our findings on faster and stronger (i.e. “preferential”) pain learning and the role of fear of pain are consistent with the biological relevance of pain and may be relevant to the development or maintenance of chronic pain.

## Introduction

To predict and control future events, we have to identify cues that precede the event and revise the learned cue-outcome association when the cue is no longer predictive. While we can learn from all kinds of observed cue-outcome couplings, some impact our learning more than others. For instance, aversive outcomes are learned faster (i.e. fewer observations are needed to form cue-outcome associations), and unlearned more slowly (i.e. more observations are needed to unlearn the association) than learning from positive experiences (van der Schaaf et al. 2022). Experimental learning studies therefore often use painful stimuli to drive learning. However, pain is not only aversive. It is also uniquely linked to threat and potential harm, which might accelerate acquisition learning and slow down extinction in comparison with equally aversive but non-painful stimuli. Fear of pain (or injury) is considered a fundamental type of fear similarly to fear of the unknown and fear of negative evaluation (Carleton 2016). Pain-related learning might therefore differ from learning from other equally aversive experiences due to its higher biological relevance, i.e. pain-predicting cues might be learned “preferentially,” that is faster, stronger or even both, than cues predicting other equally aversive experiences. However, little is known regarding such differences beyond the general effect of aversiveness. Although first studies have compared pain with other unpleasant stimuli (Fazeli and Büchel 2018; Sharvit et al. 2018; Liang et al. 2019; Čeko et al. 2022), only a few matched stimuli for unpleasantness (Sharvit et al. 2018; Liang et al. 2019).

First evidence for the distinct role of pain in threat learning stems from a recent associative learning study that compared a painful visceral stimulus with an unpleasant but non-painful loud tone as unconditioned stimuli (US; Koenen et al. 2021). Koenen and colleagues found that conditioned responses were stronger for the visceral US than the equally unpleasant loud tone. These behavioral differences were accompanied by activation differences within the posterior insula and the midcingulate cortex (MCC). Further evidence for an influence of US threat comes from studies comparing two painful USs of (presumably) different threat levels (Koenen et al. 2017, 2021; Benson et al. 2019; Schmidt et al. 2020). Healthy individuals showed a stronger propensity to acquire and reinstate US-CS associations for painful heat stimuli applied to the face as compared to the hand, although US were matched in pain intensity (Schmidt et al. 2020). Similar findings have been reported when comparing learning related to visceral versus less threatening somatic painful US (Koenen et al. 2017, 2018, 2021; Benson et al. 2019). Together, these studies suggest that individuals preferentially acquire memory for more salient or threatening stimuli. While neural mechanisms underlying fear learning and fear extinction in general have extensively been investigated and core brain circuits have been identified (see Sehlmeyer et al. 2009; Milad and Quirk 2012; Fullana et al. 2016, 2018 for review), there has been surprisingly little research on the neural underpinnings of acquisition and extinction learning in the context of pain compared to other stimuli.

Here, we tested whether cue-pain associations are acquired faster and extinguished slower than cue associations with an equally unpleasant but non-painful stimulus. We used a differential conditioning paradigm where somatic heat pain stimuli and unpleasantness-matched auditory stimuli were applied as US. Geometrical figures served as CS. Two figures were partially reinforced by either a painful US or an auditory US with a 75% reinforcement rate. A third figure served as a safety signal and was never followed by a US. Healthy volunteers rated CS valence and US-CS contingency while brain activity was assessed using functional magnetic resonance imaging (fMRI). Due to the inherent threat value of pain, we expected (i) steeper acquisition slopes indicating faster acquisition learning and (ii) flatter slopes during extinction learning for CS predicting pain (indicating slower extinction) than for CS predicting an unpleasantness matched aversive tone. This pattern is assumed to be reflected in differential CS-induced activation of brain regions involved in fear learning and salience detection (e.g. amygdala (Johnson and LeDoux 2004; Krabbe et al. 2018; Wen et al. 2022), insula (e.g. Fullana et al. 2016)). We further expected that pain-related learning would scale with stimulus-specific fear on the behavioral and neural levels.

## Materials and methods

### Subjects

Forty-five healthy volunteers were recruited locally to participate in this MR study. Inclusion criteria comprised age >18 and <60 years, normal or corrected-to-normal eyesight, right-handedness, and fluency in German. Exclusion criteria were an acute infection, a history of recurrent or chronic pain, neurological or psychiatric disorders or diabetes, ongoing participation in pharmacological intervention trials, contraindication for MR-assessment, pregnancy or breast-feeding, regular consumption of recreational drugs, regular medication intake or intake of pain medication or alcohol consumption within the past 24 h (all assessed via self-report).

Out of the 45 recruited participants, seven participants had to be excluded prior or during the experiment due to the following reasons: distortions due to strong head movements (*n* = 2), positive pregnancy test (*n* = 1), premature termination of the experiment because the MR was required for an emergency clinical scan (*n* = 1), the participant felt claustrophobic (*n* = 1), delayed information was obtained about the use of antidepressant medication (*n* = 1), and about a knee implant (*n* = 1). Thus, behavioral data of 38 right-handed participants were included in the behavioral analyses (age in years: 24.7 ± 3.8 (*M* ± SD), 20 males, 18 females). Due to technical problems, extinction data of one additional participant were lost resulting in *N* = 37 complete data sets for analyses of the extinction learning phase. Imaging data from two participants were lost due to data transfer problems. All imaging analyses are therefore based on datasets of *N* = 36 participants. The sample size estimation was based on previous learning studies using fMRI and unpleasantness-matched stimuli that have been conducted by our and other groups (Forkmann et al. 2013). This includes a study on visceral pain that used the same paradigm in *n* = 33 participants and produced robust activations (Koenen et al. 2021).

The study was conducted in accordance with the Declaration of Helsinki and had been approved by the local Ethics Committee (University of Duisburg-Essen, Germany). All participants gave written informed consent to participate and were free to withdraw from the study at any time. They received a small monetary compensation (€37.50) for their study participation.

### Experimental paradigm and procedures

Prior to any experimental procedure, participants filled in several questionnaires to obtain demographic information and assess general psychological variables (e.g. depression, anxiety) and pain-related psychological processing (e.g. pain catastrophizing, see Questionnaires). Subsequently, participants were positioned inside the MR scanner and a resting state scan was performed (data will be reported elsewhere). Following preparatory procedures that included determining detection and unpleasantness thresholds for heat and auditory stimuli and matching painful heat and auditory stimuli for unpleasantness (for details see below), participants provided fear ratings for both stimuli before the conditioning experiment commenced.

### Differential conditioning paradigm

**Fig. 1:**
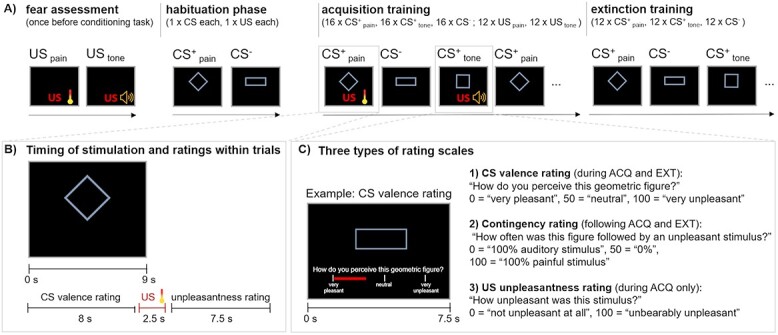
Differential conditioning paradigm. A) The experimental paradigm consisted of 3 experimental phases (habituation phase, acquisition training (ACQ), extinction training (EXT)). Assignment of the geometrical figures to experimental conditions was randomized (example shown here). In the habituation phase, each CS and US presentation was presented once, followed by valence and unpleasantness ratings for each CS and US, respectively. During acquisition training, 16 CS were presented of each CS type, of which 12 were reinforced. CS valence ratings and US unpleasantness ratings were provided on a visual analogue scale (VAS, 0–100) during every fourth CS and after every fourth US, resp. During extinction training, 12 CS of each type were shown (all unreinforced). Before the start of the conditioning experiment, participants provided ratings of their fear of the upcoming thermal and auditory stimulation. B) CS valence ratings were provided during every fourth CS. CS were presented for 9 s; US presentation was set to 2.5 s; CS^+^-US overlap was 1 s. C) VAS were displayed for 7.5 s (valence rating, US unpleasantness rating) or 15 s (contingency rating). ITI was jittered between 6 and 11 s.

We used an established differential conditioning paradigm (Schmidt et al. 2020; Schlitt et al. 2021), in which geometrical figures served as visual cues that predicted the delivery (CS^+^_pain_ or CS^+^_tone_) or absence (CS^−^) of an unconditioned stimulus (US; see Fig. 1A). Individually calibrated heat pain stimuli and an aversive tone that was matched to the heat pain stimuli in unpleasantness served as US (US_pain_ and US_tone_).

In the initial *habituation phase*, participants were familiarized with the CS and US. Each CS and US was presented once (pseudo-randomized presentation, duration: CS = 9 s, US = 2.5 s) and participants were asked to rate CS valence and US unpleasantness on a visual analogue scale (VAS) during CS presentation or following the offset of the US, respectively (see below for details). Prior to the *acquisition training*, participants were only informed about a potential association between the geometric figures and an aversive stimulation but not about the exact contingency rates or the different experimental phases (cf. Schlitt et al. 2021). During acquisition training, 16 CS^+^_pain_, 16 CS^+^_tone_, and 16 CS^−^ were presented in a pseudo-randomized order, in which (i) the same condition was presented no more than three times in a row, (ii) an equal number of each CS type was presented in the first and second half of this experimental phase, and (iii) the first and last CS^+^ trials were always reinforced. CS^+^_pain_ and CS^+^_tone_ were paired with a painful or auditory US, respectively, at a reinforcement rate of 75%. Thus, 24 US (12 CS^+^_pain_, 12 CS^+^_tone_) were presented during acquisition training. CS^−^ were never followed by an aversive event. CS^+^ were presented exclusively for 8 s before a US was presented (i.e. US and CS overlapped for 1 s; see Fig. 1B). During *extinction training*, 12 CS of each type were presented in a pseudo-randomized order (randomization restrictions see section Acquisition training). No US was applied during extinction. In all phases, inter-trial-intervals were jittered between 6 and 11 s, in which a white fixation cross was presented. To track learning dynamics of threat and safety learning, we assessed CS valence ratings repeatedly throughout the experimental phases (see below for details and Fig. 1C).

#### Stimuli

The software *Presentation* (http://www.neurobs.com) was used to present visual and auditory stimuli, to trigger the application of thermal stimuli and to record behavioral data.

##### Visual stimuli

The geometrical figures that served as CS (square, rectangle, rhombus) were presented on a projection screen located behind the MR scanner, which could be seen via a mirror attached to the head coil. The figures (RGB code: 142, 180, 227) had softened edges and were overlaid on a black background (visual angles: square—4.99° × 4.99°, rectangle—8.3° × 3.14°, rhombus—7.38° × 5.36°). The assignment of geometrical figures to CS^−^, CS^+^_pain_, or CS^+^_tone_ was pseudo-randomized across participants.

##### Heat pain stimuli

Painful heat stimuli were applied using an MR-compatible thermal device (Pathway model CHEPS, MEDOC, Israel, http://www.medoc-web.com) together with a CHEPS thermode (27 mm diameter) that was attached to the middle of the left inner forearm using an elastic tape (approximately 10 cm from the wrist). Total stimulus duration was 2.5 s (i.e. duration at target temperature varied depending on the individual target temperature). Heating and cooling rates were set to maximum (70 and 40 °C/s, respectively, baseline temperature was 35 °C) to rapidly reach the individual temperature corresponding to an unpleasantness rating of VAS 70 on a 0–100 visual analogue scale (VAS; see Fig. 1 and Outcome measures).

##### Auditory stimuli

The aversive tone was created using the software *Audacity 1.3.10-beta* (http://www.audacity.sourceforge.net/). The tone had a sawtooth waveform profile with a frequency of 1 kHz and a total duration of 2.5 s (fading in: 180 ms, fading out: 300 ms; see Forkmann et al. 2013). It was binaurally presented using MR-compatible headphones. The volume was individually adjusted to match the unpleasantness of the heat pain stimulus (see section MR assessment, US stimulus calibration, and US matching procedure). Note that the tone was intended to be unpleasant but not painful. Importantly, the characteristic that makes the tone unpleasant is not only the volume, but its sawtooth profile and frequency, which ensures that the tone is already perceived as clearly unpleasant at a moderate volume level.

#### Questionnaires

To investigate whether personality traits associated with general and pain-related psychological processing would moderate pain-related acquisition or extinction learning, participants completed the German version of the following questionnaires: “Pain Anxiety Symptoms Scale” (PASS-D, McCracken et al. (1992); German version: Walter et al. (2002)), “Pain Catastrophizing Scale” (PCS, Bishop et al. (1995); German version: Lautenbacher et al. (2009)), “Center for Epidemiologic Studies—Depression Scale” (CES-D, Radloff (1977); German version: Hautzinger and Bailer (1993)). All questionnaires were analyzed according to their respective manuals. Descriptives are given in Table S1.

### Outcome measures

Prior to the actual experiment, participants rated their level of fear of the upcoming thermal and auditory stimulation using a visual analogue scale (VAS) with the endpoints 0 = “not afraid at all” and 100 = “extremely afraid” (instruction: “How fearful are you with respect to the forthcoming pain stimuli?” and “How fearful are you with respect to the forthcoming tones?”). To track the temporal dynamics of pain-related (CS^+^_pain_) and tone-related (CS^+^_tone_) threat learning, and in particular potential differences in learning between the two types of stimuli (e.g. CS^+^_pain_ > CS^+^_tone_), we assessed CS valence repeatedly throughout acquisition and extinction training (Schmidt et al. 2020; Schlitt et al. 2021). Within the acquisition and extinction training phases, valence ratings were acquired after every fourth presentation of a CS of the same type (i.e. 4 and 3 valence ratings per CS type for acquisition and extinction training, respectively) using a 0–100 VAS with the question “How do you perceive this geometric figure?” (verbal anchors: 0 = “very pleasant,” 50 = “neutral,” 100 = “very unpleasant”). Furthermore, CS valence ratings were provided for each CS presented during the habituation phase (i.e. one valence rating per CS type). The VAS was presented as a red bar positioned below the CS (see Fig. 1C) with the cursor placed at a random starting position between VAS 25 and VAS 75. Participants moved the bar using an MR-compatible response device with their index and middle fingers of their right hand. Valence ratings had to be provided within 7.5 s.

US unpleasantness ratings were provided after every fourth presentation of a US of the same type during acquisition training (3 US unpleasantness ratings for each US type) in order to test whether the previously matched pain and tone USs were indeed comparable with respect to unpleasantness. Ratings on a 0–100 VAS (verbal anchors: 0 = “not unpleasant at all” and 100 = “unbearably unpleasant”) were prompted by the question “How unpleasant was this stimulus?”. Unpleasantness ratings had to be provided within 7.5 s.

Asking participants to repeatedly rate valence and unpleasantness was intended to track learning over the course of the experiment. However, the rating procedure leads to a “contamination” of the BOLD signal with movement-related artifacts and introduces cognitive processes unrelated to those of interest. To balance the need for a sufficient number of rating trials with the requirement of a sufficient number of uncontaminated trials, valence ratings were obtained at the end of every fourth trial.

To capture cognitive aspects of learning, we assessed participants’ explicit awareness of CS-US associations at the end of acquisition training and extinction training. To this end, we presented a VAS for each CS (CS^−^, CS^+^_pain_, CS^+^_tone_) after each experimental phase. The CS was presented above the VAS and the question “How often was this figure followed by an unpleasant stimulation?” The verbal anchors of the VAS were labeled as follows: 0 = “100% auditory stimulation”, 50 = “no unpleasant stimulation”, and 100 = “100% painful stimulation”. Again, the starting position of the VAS varied randomly between VAS 25 und VAS 75 and participants were given 15 s to provide US-CS contingency ratings.

#### MR assessment, US stimulus calibration, and US matching procedure

To minimize head movement during scanning, the head was fixated using inflatable pads on both sides of the head. Before applying any painful or auditory stimulus, we acquired resting state MRI data in each participant (data not shown here). Task-based fMRI data were acquired during all phases of the differential conditioning paradigm (see section fMRI data acquisition and analysis).

First, the individual *heat pain threshold* at the site of stimulus application was assessed using the method of limits (Fruhstorfer et al. 1976). Heat stimulation was increased by 1 °C/s, starting at a baseline temperature of 35 °C, until the participants indicated a first painful sensation by pressing a button. The average temperature of three trials was defined as the individual heat pain threshold. The upper temperature limit was set to 50 °C to avoid tissue damage. Next, auditory stimuli were presented with increasing volume until a switch from loud to unpleasantly loud stimulation was indicated by a button press. This procedure was repeated three times and the average volume level at which tones were perceived as unpleasant was calculated. During the subsequent *temperature calibration* and *matching procedures* (see below), we ran an EPI sequence to create an acoustic background identical to the actual conditioning experiment. To determine the individual temperature level corresponding to an unpleasantness level of 70 on the 0–100 VAS (i.e. *temperature calibration*), participants were presented with heat stimuli of varying temperature levels around their individual heat pain threshold (range: −1 °C < pain threshold <+3.5 °C, temperature difference 0.5 °C, each temperature level was applied twice). Participants rated the unpleasantness of each stimulus on a VAS. The temperature corresponding to an unpleasantness level of VAS 70 was calculated from the ratings using linear regression analysis in R (R Core Team 2020). Finally, we applied a *matching procedure* in order to identify heat levels and volume levels that induced the same level of unpleasantness for heat and tone stimuli (see Forkmann et al. 2013). To this end, the individually calibrated heat pain stimulus (VAS 70) was presented and immediately followed by an auditory stimulus. Participants indicated by button press whether the tone was more, less, or equally unpleasant than the heat stimulus. Depending on the participants’ response, the volume was automatically reduced or increased in the subsequent trial. In case the participant indicated equal unpleasantness for tone and pain stimuli, the matching trial stopped. This procedure was performed five times. The individual volume level corresponding to the VAS 70 pain stimulus was calculated as the mean of five matching trials. The preparatory procedures ended with a short *test phase* in which three aversive stimuli of each type were presented and participants provided unpleasantness ratings to ensure comparable unpleasantness ratings for both US. This procedure was repeated with slightly altered volume levels if the unpleasantness of tone and pain stimuli was not comparable.

Following the differential conditioning paradigm, a high-resolution anatomical MR (T1^*^-weighted) was obtained.

### Data analysis—behavior

The software package R version 1.3.1073 (R Core Team 2020) was used to analyze behavioral data.

#### US unpleasantness

To test whether stimulus unpleasantness during habituation and acquisition training differs significantly for both types of US, the 4 ratings for each US (1 during habituation, 3 during acquisition) were averaged for each participant. Subsequently, a paired *t*-test was performed.

#### Fear ratings

We tested whether fear ratings related to pain and tone differed significantly using a Wilcoxon signed-rank test, as fear ratings for both pain and tone US were not normally distributed.

#### Valence ratings

To compare learning of CS^+^_pain_ and CS^+^_tone_, separate linear mixed model (LMM) analyses were calculated for each of the experimental phases, i.e. acquisition and extinction training. Valence ratings of the habituation phase were included as a baseline in the acquisition LMM as these ratings were provided prior to any pairing of US and CS. The last valence ratings for each CS provided during acquisition training were included as a baseline rating in the extinction LMM. Linear mixed models were further used to estimate the effects of potential covariates on emotional processing (i.e. valence changes). Effect sizes were calculated using the R package *EMAtools* version 0.1.3 (Kleiman 2017).

To test for differences in learning between CS types, the factors “CS type” and “time” and their interaction were included as fixed effects into the model. Time was modeled linearly based on a formal comparison of two models; one that assumes a linear trend in valence ratings and another one assuming a quadratic trend. Comparing both models using the *anova* function in R showed that the latter model did not significantly fit better than the simpler, linear model (for acquisition training: *χ^2^*(3) = 6.21, *P* = 0.102; for extinction training: *χ^2^*(3) = 1.19, *P* = 0.76). To account for individual differences in CS valence ratings at baseline and their individual changes over time, the LMM included a random intercept of *subjects* and random slopes of the factors *CS type, time*, and *subjects*. According to the Akaike information criterion (AIC), this model best predicted the data for each phase as compared to models without random slopes for the factors *CS type* and *time*. Using a Wilcoxon signed rank test, we further tested whether participants showed complete extinction at the end of the experiment by comparing CS valence ratings provided after extinction training with baseline ratings obtained during the habituation phase. To test that the observed differences between baseline and last extinction ratings were not driven by higher starting points (i.e. more negative valence) at the end of the acquisition phase, we performed a post-hoc analysis (LMM) with the factors *CS type* and *time* (the factor time comprising only two levels (= “timepoints”), namely “1” (= habituation rating) and “2” (= last extinction rating). The last acquisition rating was included as a covariate of no interest.

To explore the potential influence of further characteristics, the following variables were added separately to the models for each experimental phase: the difference of individual fear ratings (*diff fear* = fear rating pain − fear rating tone), which were provided prior to the conditioning task, as well as the following questionnaire scores: pain catastrophizing (PCS), pain anxiety and sensitivity (PASS) and depressive symptoms (CES-D). Each potential covariate was modeled to interact with the factors *time* and *CS type* (i.e. three-way interaction, e.g. *time* × *CS type* × *diff fear*).

#### Contingency ratings

Another LMM was performed to test for differences in contingency ratings between CS types and changes in contingency ratings between experimental phases (acquisition, extinction). Due to the low number of data points, this model was calculated without random slopes and thus only comprised *CS type* and *phase* as well as their interaction as fixed effects, and a random intercept for the *subjects.*

### fMRI—data acquisition and analysis

MR scanning was performed on a 3 T MRI system (Siemens Trio) with a standard 20-channel head and neck coil. A total of 38 axial slices (slice thickness, 3 mm, slice gap 0.45 mm) per volume were acquired using a gradient EPI T2^*^-sensitive sequence with the following parameters: repetition time (TR), 2.4 s; echo time (TE), 28 ms; flip angle, 90°; generalized partially parallel acquisitions (GRAPPA) *r* = 3, field of view, 221 × 221 mm^2^. After the functional scan, a high-resolution anatomical image was obtained for each participant using a T1-weighted magnetization-prepared rapid acquisition gradient echo sequence (192 slices; slice thickness, 1 mm; TR, 2.3 s; TE, 2.07 ms; flip angle, 9°; GRAPPA *r* = 2; field of view, 256 × 256 mm^2^). Functional and anatomical data were preprocessed using the highly automated and validated preprocessing pipeline *fMRIPprep* 1.3.0.post2 (Esteban et al. 2019), which is based on *Nipype* 1.1.8 (Gorgolewski et al. 2011). Preprocessing steps will be only briefly described here. For a detailed description of anatomical and functional data preprocessing as part of *fMRIprep*, see Supplementary Methods.

#### Data preprocessing

In short, participants’ T1-weighted (T1^*^w) images were corrected for intensity non-uniformity and used as T1w-reference throughout the workflow. T1 preprocessing further included skull-stripping, spatial normalization and brain tissue segmentation into cerebrospinal fluid, white-matter, and gray-matter. For the functional data, the first six volumes were discarded to compensate for T1 saturation effects. The following preprocessing steps of the functional data were performed separately for the acquisition and extinction training phases. The BOLD reference was co-registered to the T1w reference, motion corrected, and slice-time corrected. The BOLD time-series were resampled to MNI152NLin2009cAsym standard space. For each experimental phase (i.e. acquisition, extinction), several physiological regressors were extracted to allow for component-based noise correction (“CompCor”; Behzadi et al. 2007). Principal components were estimated after high-pass filtering the preprocessed BOLD time-series with a 128 s cut-off for two CompCor variants: temporal (tCompCor) and anatomical (aCompCor). For aCompCor, 6 components are calculated from each experimental phase that were later added as nuisance regressors to the single subject design matrices for the acquisition training and the extinction training, respectively (see section fMRI data analysis). Note that these parameters are not identical with the 6 standard motion parameters estimated during the realignment procedure in SPM. Finally, functional images were smoothed with a 6 mm Gaussian kernel with FWHM using SPM12 (https://www.fil.ion.ucl.ac.uk/spm/). No participants had to be excluded due to excessive head movement (mean FD > 0.55, as recommended by Parkes et al. 2018).

#### fMRI data analysis

Data analysis was performed using the general linear model (GLM). Separate fMRI analyses were performed for acquisition and extinction training phases. To investigate brain activation during acquisition training, individual design matrices (subject level) included 5 regressors of interest that coded for the 3 CS (CS^−^, CS^+^  _pain_, CS^+^  _tone_) and 2 US (US _pain_, US _tone_). Individual design matrices for extinction training comprised 3 regressors of interest coding for the 3 CS (CS^−^, CS^+^  _pain_, CS^+^  _tone_). For both experimental phases, CS events were modeled using a stick function (0 s duration) and were convolved with a canonical hemodynamic response function (hrf). US events during acquisition training were also modeled with 0 s duration (stick function) and convolved with the hrf. For both phases, CS trials in which participants had also provided CS valence ratings (i.e. every fourth CS presentation) were treated as regressor of no interest in order to control for rating-related signal changes (modeled duration = 9 s, i.e. duration of CS rating trial). This regressor of no interest also included further rating phases (US unpleasantness ratings, modeled duration = 7.5 s). Trials in which US-CS contingency ratings had to be provided (i.e. at the end of the acquisition training or extinction training) were discarded prior to any data analysis. The single subject design matrices comprised 6 nuisance regressors (aCompCor; Behzadi et al. 2007) for each phase to remove physiological noise, including motion-related artifacts (Muschelli et al. 2014). Differential contrasts (e.g. CS^+^_pain_ > CS^+^_tone_) were calculated on the subject level and were subsequently included to a GLM on the group level, in which one sample *t*-tests were calculated. Note that because only one CS^−^ was used, the contrast (CS^+^_pain_ > CS^+^_tone_) is statistically identical to the contrast [(CS^+^_pain_ > CS^−^) > (CS^+^_tone_ > CS^−^)].

To test for brain regions showing increasing engagement during acquisition training, we calculated an additional first-level model and applied time modulation (first order, i.e. linear increase) as provided by SPM12 to the regressors modeling CS^+^_pain_, CS^+^_tone_, and CS^−^. Differential contrasts of these regressors modeling time × condition interactions (e.g. CS^+^_pain × time_ > CS^+^_tone × time_) were then entered into a second group-level GLM and one-sample *t*-tests were calculated. The same approach was performed for the extinction training phase.

Behavioral analyses revealed that individual fear ratings differed significantly between pain and aversive tone stimuli and that learning behavior was modulated by individual fear (see section Valence ratings ). To probe the neural mechanisms driving this behavioral modulation, individual differences in fear ratings (*diff fear = fear of pain − fear of tone*) were included as a covariate on the group level. A positive weight (+1) on the regressor coding *diff fear* identified brain regions showing a stronger increase in activation for CS^+^_pain_ than CS^+^_tone_ during acquisition training (i.e. time × condition, see above) that scaled with differences in fear ratings (i.e. the stronger the neural activity the more fearful participants were of pain in comparison with aversive tone stimuli).

Group-level analyses were limited to anatomically defined regions-of-interest (ROI), including brain areas belonging to the salience, acquisition, and extinction networks (Quirk and Mueller 2008; Sehlmeyer et al. 2009; Menon and Uddin 2010; Wiech et al. 2010; Fullana et al. 2016, 2018; Ernst et al. 2019). Anatomical ROIs comprised the insula, the cingulate cortex, amygdala, hippocampus, the ventromedial prefrontal cortex (vmPFC; Mackey and Petrides 2014), and the cerebellum. We further included anatomical ROIs for brain regions known to be associated with pain and auditory processing (parietal operculum, thalamus, putamen, parahippocampal gyrus, Heschl gyrus). All anatomical ROIs were defined as unilateral, binary masks based on the Harvard–Oxford cortical and subcortical structural atlases (Desikan et al. 2006) or the MIST atlas (Urchs et al. 2019) in the case of the cerebellum. All reported imaging results are FWE corrected values with a *P*-level of *P* < 0.05. When comparing both US (e.g. US_pain_ > US_tone_; included as a validity check only), we report whole brain results (whole-brain FWE corrected, *P* < 0.05). Cluster size is only reported for this analysis. For every contrast regarding CS-related activity differences, FWE correction was performed via small volume correction (SVC) for each a priori defined region of interest. In addition to the SVC results, we also report uncorrected data with a *P*-level of *P* < 0.001 and cluster size *k* > 10 for transparency and to enable future meta-analyses of the data. The results and discussions focus on ROI analyses only. Please note that the terms “pain-specific” and “tone-specific” will be used in the results section to refer to analyses and results that directly compare learning from these two stimuli. However, our analyses do not allow for conclusions regarding pain specificity in the strict sense (see section Discussion). Furthermore, this study focused on the direct comparison of pain-predicting and tone-predicting cues. For transparency, we also report results related to the comparison of each CS^+^ with the CS^−^. Results are given in the Supplement (Tables S3–S6).

## Results

### Heat pain threshold and stimulation during the conditioning experiment

On average, the heat pain threshold was 44.25 °C ± 2.33 [40.3–50] (M ± SD, [range]) and the temperature applied during the conditioning experiment that corresponded to VAS 70 was 47.05 °C ± 1.68 [43.5–49.8]. On average, the volume of the aversive tone corresponding to the unpleasantness of the heat pain stimulus was 91.5 ± 15.3 dB SPL (due to technical failure, data of *N* = 11 participants are missing).

### Fear ratings

Fear ratings for unpleasantness-matched heat and tone stimuli provided before the conditioning task differed significantly (*V* = 455.5, *P* < 0.001) with participants being more fearful of pain (fear of pain: 25.63 ± 23.53 [0–73]; fear of tone: 13.34 ± 11.48 [0–40]. Due to this significant difference, individual differences in fear ratings (*diff fear*) were included as a covariate into the LMM (see section Data analysis—behavior).

### Unpleasantness ratings

As intended, mean unpleasantness ratings during habituation and acquisition did not differ significantly between US_pain_ (55.53 ± 14.09) and US_tone_ (50.80 ± 15.35; *t*(37) = 1.70, *P* = 0.10). US-specific unpleasantness ratings across the habituation and acquisition phase are shown in Fig. S1.

### Valence ratings

#### Habituation phase

Baseline CS valence ratings did not differ significantly between CS types (*M* ± SD, CS^+^_pain_: 37.21 ± 17.45, CS^+^_tone_: 41.82 ± 18.62, CS^−^: 37.29 ± 19.33, *F*(2, 74) = 1.18, *P* = 0.31, *η^2^_p_* = 0.03), indicating that CS were comparable in valence prior to conditioning.

#### Acquisition training

**Fig. 2:**
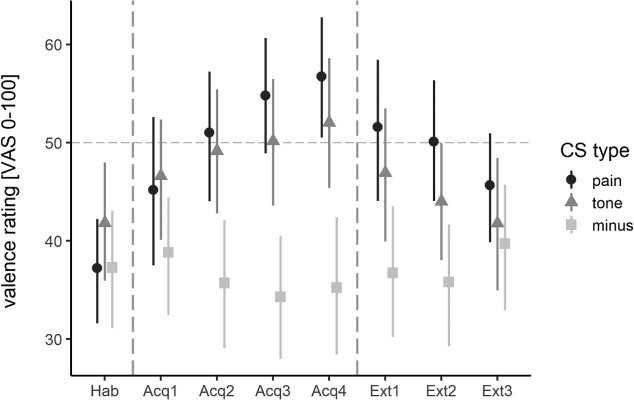
Mean valence ratings [VAS 0–100] provided during the 3 experimental phases (habituation phase, Hab; acquisition training, Acq1–Acq4; extinction training, Ext1–Ext3) for each CS type (CS^−^, square; CS^+^_pain_, circle; CS^+^_tone_, triangle). VAS anchors were labeled as 0 = “very pleasant”, 50 = “neutral”, 100 = “very unpleasant.” Neutral valence is marked with a dotted horizontal line. Ratings above the dotted line represent negative valence, ratings below represent positive valence. Experimental phases are separated by vertical dashed lines. Error bars indicate 95% confidence intervals. For single data see Fig. S2).

LMM analyses revealed differences in acquisition learning between CS^+^_pain_, CS^+^_tone_, and CS^−^ as indicated by a significant interaction of *CS type* and *time* (*F*(2, 416) = 27.95, *P* < 0.001, *η^2^_p_* = 0.12; see Fig. 2; for single data please see Fig. S2). CS^+^_pain_ valence ratings showed a steeper increase (i.e. steeper slope) in negative valence during acquisition (β *=* 4.87, SE = 0.62) than CS^+^_tone_ (β = 2.40, SE = 0.62; *t*(416.00) = 3.20, *P* = 0.001, *d* = 0.31) and CS^−^ (β = −0.87, SE = 0.63; *t*(416.00) = 7.45, *P* < 0.001, *d* = 0.73). Moreover, valence ratings for CS^+^_tone_ showed a significantly steeper increase in negative valence than CS^−^ valence ratings (*t*(416.00) = 4.25, *P* < 0.001, *d* = 0.43). Interestingly, CS^−^ valence ratings did not significantly change during acquisition training (*t*(138.35) = −1.39, *P* = 0.17).

**Fig. 3:**
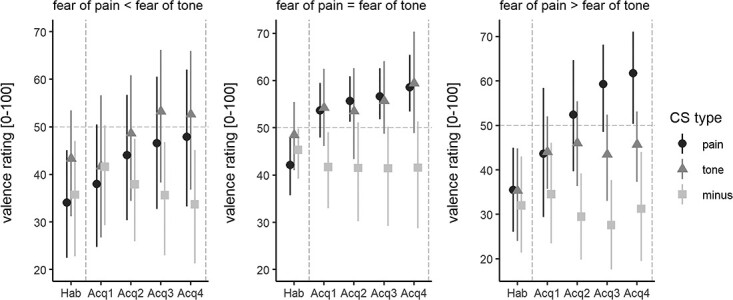
Modulation of CS valence by individual fear of pain and fear of tone. The figure depicts valence ratings (means and 95% confidence intervals) provided during habituation (Hab) and acquisition training (Acq1–Acq4) for each CS type (CS^−^, CS^+^_pain_ or CS^+^_tone_) illustrated separated for individuals reporting lower pain-related fear than tone-related fear (left, *n* = 11), similar or slightly higher pain-related fear ratings (middle, *n* = 15; diff fear pain-fear tone = 0–10) and higher pain-related than tone-related fear (right, *n* = 12). For illustration purposes only, participants were separated into three groups according to the difference in fear ratings to reach approximately similar subsample sizes.

Since fear ratings differed significantly between painful and auditory stimuli, the difference in fear ratings was added to the model as a covariate (fixed effect) to test for its modulatory effect, which increased model fit significantly (ΔAIC = −2.6, *P* = 0.024). A significant interaction of *CS type* × *time* × *diff fear* during acquisition training was found (*F*(2, 414) = 4.65, *P* = 0.01, *η^2^_P_* = 0.02), implying that differences in pain- and tone-related fear indeed modulated differences in pain- and tone-related acquisition learning. Post hoc tests revealed that greater fear of pain than fear of tone was associated with a steeper increase in negative valence for CS^+^_pain_ as compared to CS^+^_tone_ (β = 0.09, SE = 0.04, *t*(414.00) = 2.28, *P* = 0.02, *d* = 0.22; for visualization purposes see Fig. 3).

To test whether the observed difference in pain-related and tone-related acquisition learning was driven by differences in individual fear ratings, we built an additional model with *diff fear* as a covariate of no interest. This analysis showed that, when accounting for intra-individual differences in fear, threat learning was still enhanced for CS^+^_pain_ than CS^+^_tone_ (β = 2.46, SE = 0.77, *t*(416.00) = 3.203, *P* = 0.001).

Although overall, the two US had successfully been matched for unpleasantness (see above), we decided to include differences in mean US unpleasantness (unpleasantness US_pain_-US_tone_) in the LMM, which increased model fit significantly (ΔAIC = −20.7, *P* < 0.001). Even when accounting for intra-individual differences in US unpleasantness, threat learning was still significantly stronger for CS^+^_pain_ than CS^+^_tone_ (β = 2.46, SE = 0.77; *t*(416.00) = 3.20, *P* = 0.001, *d* = 0.31). None of the other covariates (sex, PCS, subscales of the PASS, CES-D; questionnaire data is given in Table S1) improved model fit significantly (all *P* > 0.5), indicating that they did not substantially modulate acquisition learning in our study.

#### Extinction training

As in the case of acquisition learning, the significant interaction indicated a difference in the temporal development of ratings between the three CS (interaction of *CS type* and *time* (*F*(2, 294) = 18.24, *P* < 0.001, *η^2^_p_* = 0.11)*.* Importantly, this interaction was driven by a significant difference between the CS^−^ and each CS^+^ (CS^−^ vs. CS^+^_pain_: β = −5.11, SE = 0.91, *t*(294) = −5.62, *P* < 0.001, *d* = −0.66; CS^−^ vs. CS^+^_tone_: β = −4.29, SE = 0.91; *t*(294) = −4.72, *P* = 0.001, *d* = −0.55), whereas the slopes of the two CS^+^ were not significantly different (β = 0.81, SE = 0.91; *t*(294.00) = 0.90, *P* = 0.37, *d* = 0.11). However, negative valence ratings for both CS^+^ decreased significantly during extinction training (CS^+^_pain_: β = −3.99, SE = 0.94, *t*(74.58) = −4.26, *P* < 0.001, *d* = −0.99; CS^+^_tone_: β = −3.17, SE = 0.94; *t*(74.58) = −3.39, *P* = 0.001, *d* = −0.78), while valence ratings for the CS^−^ did not change significantly (β = 1.12, SE = 0.94; *t*(74.58) = 1.206, *P* = 0.23, *d* = 0.28).

Interestingly, at the last extinction trial valence ratings for the CS^+^_tone_ (*V* = 341, *P* = 0.46) and CS^−^ (*V* = 170, *P* = 0.31) had returned to baseline (i.e. during the habituation phase at the beginning of the experiment) whereas ratings for the CS^+^_pain_ were still significantly more negative compared to the beginning (*V* = 134.5, *P* = 0.009). This finding was not driven by the higher starting point (i.e. stronger negative valence rating) for CS^+^_pain_ than CS^+^_tone_ at the last acquisition rating. A post hoc analysis showed that, when including the last acquisition rating as a covariate of no interest, valence ratings for CS^+^_pain_ were significantly more negative after extinction training than at habituation (CS^+^_pain_: β = 8.58, SE = 2.78; *t*(97.72) = 3.09, *P* = 0.003), while valence ratings for the CS^+^_tone_ and CS^−^ did not differ between habituation and the end of extinction training (CS^+^_tone_: β = −0.03, SE = 2.79; *t*(98.15) = −0.009, *P* = 0.99; CS^−^: β = 2.62, SE = 2.82; *t*(99.74) = 0.94, *P* = 0.35).

The inclusion of individual differences in fear (*diff fear*) or differences in mean US unpleasantness as covariates of no interest did not improve model fit (diff fear: ΔAIC = −1.6, *P* = 0.11; difference in mean US unpleasantness: ΔAIC = −1.4, *P* = 0.07), indicating that these variables did not significantly modulate extinction learning. Furthermore, none of the other tested covariates (sex, PCS, subscales of the PASS, CES-D) improved model fit significantly (all *P* > 0.29), which suggests that they did not substantially modulate extinction learning in this study sample.

### Contingency ratings

Differences in contingency ratings were analyzed using LMM. Raw contingency ratings were transformed as described in the methods. The best fitting model included *phase* and *CS type* as fixed effects and *subjects* as random effect (AIC = 2273.3). This analysis revealed significant main effects and interactions for contingency ratings provided after acquisition and extinction training (ME *phase: F*(1, 185) = 15.21, *P* < 0.001*,* ME *CS type: F*(2, 185) = 17.76, *P* < 0.001), IA *CS type* × *phase: F*(2, 185) = 6.98*, P* = 0.001; see Fig. S3. In detail, contingency ratings for CS^+^_pain_ (β = 50.26, SE = 5.66) were significantly higher than for CS^+^_tone_ (β = 25.47, SE = 5.66; *t*(185) = 3.24, *P* = 0.001, *d* = 0.79) and CS^−^ (CS^−^: β = 0.16, SE = 4.63; *t*(185) = 6.54, *P* < 0.001, *d* = 1.51) after acquisition training. After extinction training, contingency rating were comparable between CS^+^_pain_ (β = 9.90, SE = 5.66) and CS^−^ (β = −2.63, SE = 5.66; *t*(185) = 1.63, *P* = 0.10, *d* = −0.38), as well as CS^+^_pain_ and CS^+^_tone_ (β = 16.90, SE = 5.66; *t*(185) = 0.91, *P* = 0.36, *d* = 0.21). However, contingency ratings for CS^+^_tone_were significantly higher than for CS^−^ (*t*(185) = 2.55, *P* = 0.01, *d* = 0.59). The decrease of contingency ratings from after acquisition training to after extinction training was significantly steeper for CS^+^_pain_ (β = −40.37, SE = 7.66) compared to CS^+^_tone_ (β = −8.58, SE = 7.66; *t*(185) = −2.94, *P* = 0.004, *d* = −0.71).

### Imaging results

#### Acquisition training—CS-related brain activation

BOLD-responses to pain-predicting cues (CS^+^_pain_) were compared to BOLD responses to cues predicting an aversive tone (CS^+^_tone_) during acquisition training (results see Table 1). When considering the entire acquisition training phase, pain-specific activation (CS^+^_pain_ > CS^+^_tone_) was found in the left anterior insula and ventromedial prefrontal cortex (vmPFC; see Table 1). Tone-specific activation (CS^+^_tone_ > CS^+^_pain_) was found in the right thalamus, bilateral parietal operculum, and right cerebellum. Pain-specific and tone-specific neural activation elicited by US ([US_pain_ > US_tone_] and [US_tone_ > US_pain_]) as well as activation differences between CS^+^_pain_ or CS^+^_tone_ and CS^−^ are given in Tables S2 and S3.

**Table 1:** Modality-specific neural responses to cues (CS^+^) predicting pain versus aversive tone during acquisition training.

Contrast	Region	MNI-coordinates				*T*	*P*
		H	*x*	*y*	*z*		
*Differential contrasts (CS^+^)*							
CS^+^_pain_ > CS^+^_tone_							
	Insula, anterior	L	−39	8	−6	4.50	0.004^*^
	vmPFC	L	−12	44	5	4.34	0.021^*^
CS^+^_tone_ > CS^+^_pain_							
	Thalamus	R	14	−16	19	4.77	0.005^*^
	Parietal operculum	L	−48	−30	15	4.52	0.002^*^
	Parietal operculum	R	44	−30	22	3.52	0.028^*^
	Cerebellum	R	32	−42	−26	5.33	0.007^*^
	*Postcentral gyrus*	*L*	*−30*	*−33*	*67*	*4.29*	*<0.001*
	*Postcentral gyrus*	*R*	*41*	*−27*	*67*	*4.20*	*<0.001*
	*Insula*	*L*	*−30*	*−22*	*19*	*4.17*	*<0.001*
	*Precentral gyrus (primary motor cortex)*	*R*	*3*	*−25*	*60*	*4.14*	*<0.001*
	*Superior parietal lobe*	*L*	*−27*	*−57*	*67*	*4.13*	*<0.001*
	*Precentral gyrus*	*R*	*53*	*−10*	*50*	*4.06*	*<0.001*
	*Lateral occipital cortex*	*L*	*−21*	*−75*	*50*	*3.94*	*<0.001*

Peak voxels indicate significant activation after small volume correction using pre-defined ROIs (*P*_FWE_ < 0.05, ^*^) or whole-brain analyses (in italic font, cluster size *k* ≥ 10; all *P* < 0.001 uncorrected), respectively. Exact unilateral *P*-values are provided. For activation maps see Fig. S5*.* Abbreviations: CS, conditioned stimulus; H, hemisphere; L, left; R, right.

#### Activity changes during acquisition training and association with individual fear ratings

**Fig. 4:**
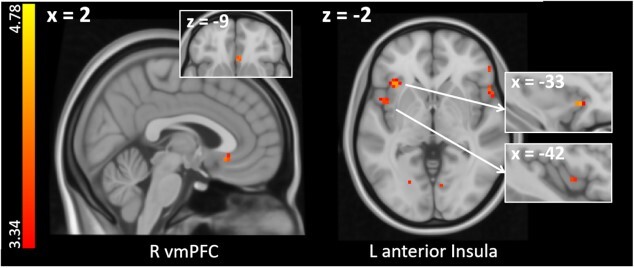
Time × condition interaction: Increasing pain-specific neural activation during acquisition training (CS^+^_pain × time_ > CS^+^_tone × time_). Significant neural activations are superimposed on a structural T1-image and thresholded at *P* < 0.001, *k* > 0, uncorrected for visualization purposes; color bar indicates *t*-scores.

To further explore the temporal nature of pain-related acquisition, we investigated *time* × *condition* interactions to identify brain regions that showed a linear increase in activation over the course of the acquisition training (see section fMRI data analysis). This analysis revealed an increased involvement of the left anterior insula for pain-predicting CS than for tone-predicting CS (CS^+^_pain × time_ > CS^+^_tone × time_; see Fig. 4 and Table 2). No significant results were found for the reverse contrast (CS^+^_tone × time_ > CS^+^_pain × time_).

**Table 2:** Time × condition interaction: Modality-specific neural responses to cues (CS^+^) predicting pain versus tone and showing increasing recruitment during acquisition training.

Contrast	Region	MNI-coordinates				*T*	*P*
		H	*x*	*y*	*z*		
*Time by condition interaction*							
CS^+^_pain × time_ > CS^+^_tone × time_							
	Insula, anterior	L	−33	20	−2	4.15	0.013^*^
	Insula, anterior	L	−42	5	−2	3.7	0.046^*^
	*Temporal pole*	*R*	*47*	*8*	*−30*	*4.51*	*<0.001*
	*Frontal pole*	*L*	*−39*	*53*	*1*	*4.16*	*<0.001*
	*Frontal pole*	*R*	*23*	*47*	*19*	*4.09*	*<0.001*
	*vmPFC*	*R*	*3*	*23*	*−9*	*4.00*	*<0.001*
CS^+^_tone × time_ > CS^+^_pain × time_							
	–	–	–	–	–	–	–
*Modulation by individual fear ratings*							
(CS^+^_pain × time_ > CS^+^_tone × time_) × diff fear							
	Amygdala	R	26	2	−23	3.73	0.025^*^
	Pallidum	L	−21	−7	−2	3.41	0.045^*^

Peak voxels indicate significant activation after small volume correction using pre-defined ROIs (*P*_FWE_ < 0.05, ^*^) or whole-brain analyses (in italic font, cluster size *k* ≥ 10; all *P* < 0.001 uncorrected), respectively. Exact unilateral *P*-values are provided. Extended results pertaining increasing modality-related neural responses to cues predicting pain (CS^+^_pain_ > CS^−^) or tone (CS^+^_tone_ > CS^−^) during acquisition training and their modulation by modality-specific fear ratings are given in Table S4. Abbreviations: CS, conditioned stimulus; H, hemisphere; L, left; R, right.

**Fig. 5:**
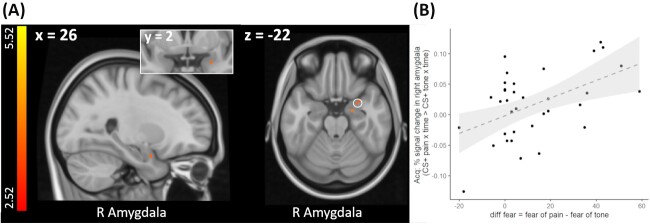
A) Increasing neural activation for pain-predicting versus tone-predicting cues during acquisition training scaling with stronger fear of pain than fear of tone as revealed by a time × condition interaction with individual differences in stimulus-related fear ratings as a covariate (i.e. [CS^+^_pain × tone_ > CS^+^_tone × time_] × diff fear). For visualization purposes, neural activations are superimposed on a structural T1-image and thresholded at *P* < 0.001, *k* = 0; color bar indicates *t*-scores. B) For visualization purposes % signal change in the right amygdala (peak voxel + 5 mm sphere) is plotted against individual differences in fear ratings (diff fear). Black dots depict single participant data, dashed line and gray area represents the regression line and 95% confidence interval. See Fig. S4 for a plot showing % signal change in the left pallidum plotted against individual differences in fear ratings (diff fear). See Fig. S6 for an overlay of the observed amygdala activation onto an anatomical mask of the right amygdala and an activation map based on an automated meta-analysis of 21 studies included in the neurosynth database under the search term “fear”.

Since behavioral differences in acquisition learning (i.e. CS valence changes) between pain- and tone-predicting CS were modulated by differences in US-related fear ratings (see section Valence ratings), we further tested whether pain-specific changes in brain activation during acquisition training (CS^+^_pain × time_ > CS^+^_tone × time_) scaled with individual fear ratings (*diff fear* as covariate of interest, see section fMRI data analysis). This analysis revealed that pain-specific activation changes in the right amygdala as well as the left pallidum were associated with individual differences in fear ratings (see Fig. 5 and Table 2).

#### Extinction training—CS-related brain activation

**Fig. 6:**
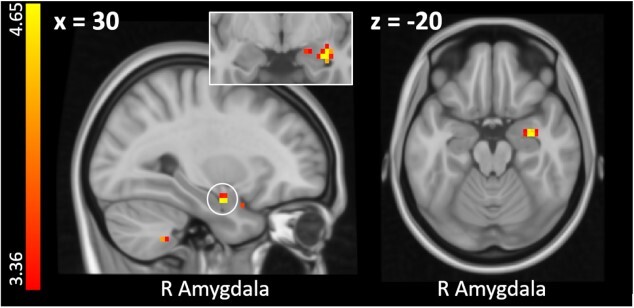
Enhanced activity within the right amygdala for pain-predicting than tone-predicting cues during extinction training for participants reporting higher fear of painful versus auditory stimulation (i.e. [CS^+^_pain_ > CS^+^_tone_] × diff fear). Neural activations are superimposed on a structural T1-image and thresholded at *P* < 0.001, *k* = 0, uncorrected for visualization purposes; color bar indicates *t*-scores. See Fig. S7 for an overlay of the observed amygdala activation onto an anatomical mask of the right amygdala and an activation map based on an automated meta-analysis of 21 studies included in the neurosynth database under the search term “fear.”

During extinction training, in which CS were never followed by US, the CS that had previously signaled an aversive tone induced greater activity in the right parahippocampal gyrus than the CS predicting pain (CS^+^_tone_ > CS^+^_pain_; see Table 3). The reverse contrast (CS^+^_pain_ > CS^+^_tone_) did not reveal significant results. However, when testing whether extinction-related activity scaled with individual differences in fear ratings, we found significant activation in the right amygdala extending into the right hippocampus (see Fig. 6 and Table 3). This indicates that participants reporting higher fear of pain than fear of tone showed residual activation in brain regions associated with fear and learning.

**Table 3:** Modality-specific neural responses to cues (CS^+^) during extinction training that predicted painful versus unpleasant auditory stimuli during acquisition.

Contrast	Region	MNI-coordinates				*T*	*P*
		H	*x*	*y*	*z*		
*Differential contrasts (CS^+^)*							
CS^+^_pain_ > CS^+^_tone_							
	–	–	–	–	–	–	–
CS^+^_tone_ > CS^+^_pain_							
	–	–	–	–	–	–	–
*Modulation by individual fear ratings*							
[CS^+^_pain_ > CS^+^_tone_ ] × diff fear							
	Amygdala	R	29	−7	−19	4.62	0.002^*^
	Amygdala/Hippocampus	R	17	−10	−16	3.7	0.026^*^

Peak voxels indicate significant activation after small volume correction using pre-defined ROIs (*P*_FWE_ < 0.05, ^*^). Exact unilateral *P*-values are provided. Extended results pertaining modality-related neural responses to cues predicting pain (CS^+^_pain_ > CS^−^) or an aversive tone (CS^+^_tone_ > CS^−^) during extinction training and their modulation by modality-specific fear ratings are given in Table S5. Abbreviations: CS, conditioned stimulus; H, hemisphere; L, left; R, right.

A stronger increase in BOLD activity over the course of the extinction phase (i.e. time by condition interaction) was observed for the right parahippocampal gyrus for the CS^+^_pain_ versus CS^+^_tone_, while the left posterior cingulate gyrus showed an increasing recruitment for CS^+^_tone_ versus CS^+^_pain_ (see Table 4).

**Table 4:** Time × condition interaction: Modality-specific increase in neural responses to cues (CS^+^) that previously predicted painful versus unpleasant auditory stimuli as activated during extinction training.

Contrast	Region	MNI-coordinates					
		H	*x*	*y*	*z*	*T*	*P*
*Time by condition interaction*							
CS^+^_pain × time_ > CS^+^_tone × time_							
	Parahippocampal gyrus, posterior	R	26	−30	−19	3.65	0.013^*^
	*Lingual gyrus*	*R*	*11*	*−78*	*−12*	*4.95*	*<0.001*
	*Intracalcarine gyrus/V1*	*R*	*26*	*−60*	*8*	*4.01*	*<0.001*
	*Intracalcarine gyrus/V1*	*L*	*−6*	*−72*	*12*	*4.53*	*<0.001*
	*Visual cortex (V4)*	*L*	*−15*	*−87*	*−9*	*3.95*	*<0.001*
CS^+^_tone × time_ > CS^+^_pain × time_							
	Cingulate gyrus, posterior	L	−3	−45	29	3.78	0.033^*^
*Modulation by individual fear ratings*							
[CS^+^_pain × time_ > CS^+^_tone × time_ ] × diff fear							
	–	–	–	–	–	–	–

Peak voxels indicate significant activation after small volume correction using pre-defined ROIs (*P*_FWE_ < 0.05, ^*^) or whole-brain analyses (in italic font, cluster size *k* ≥ 10; all *P* < 0.001 uncorrected), respectively. Exact unilateral *P*-values are provided. Extended data pertaining changes in modality-related neural responses to cues that have previously predicted pain (CS^+^_pain_ > CS^−^) or tone (CS^+^_tone_ > CS^−^) and their modulation by modality-specific fear ratings are given in Table S6. Abbreviations: CS, conditioned stimulus; H, hemisphere; L, left; R, right.

## Discussion

This study aimed to elucidate behavioral and neural mechanisms of acquisition and extinction learning related to pain as opposed to an equally unpleasant tone. Healthy participants performed a classical conditioning task with painful heat stimuli as one CS^+^ (CS^+^_pain_) and an unpleasantness matched tone as the second CS^+^ (CS^+^_tone_). Behavioral results show enhanced acquisition learning for the CS predicting pain compared to tone. This finding was accompanied by stronger activity in the left anterior insula and vmPFC during CS^+^_pain_ presentation, which moreover increased over the acquisition phase. This difference in acquisition learning was modulated by pain- and tone-related fear. Participants reporting higher fear of pain than tone demonstrated enhanced learning from pain than tone, which was reflected in stronger activation of the right amygdala and left pallidum. While extinction learning did not significantly differ between the two (now unreinforced) CS on the behavioral level, the CS that had previously signaled pain induced greater activity in the right parahippocampal gyrus. Enhanced fear of pain was again associated with higher activity in the right amygdala during CS^+^_pain_ than CS^+^_tone_ presentation during extinction training.

### Behavior

Our findings of enhanced acquisition learning for pain-predicting cues underscore previous observations of differences in learning depending on the threat value of the conditioned stimulus (Schmidt et al. 2020; Koenen et al. 2021). So far, preferential pain learning and the modulatory role of pain-related cognitions have only sparsely been investigated, and often the methods and experimental designs did not allow for the exploration of pain-specific aspects of learning (Neumann and Waters 2006; Weike et al. 2008). By carefully matching the unpleasantness of a painful heat stimulus and a loud tone and comparing learning on the behavioral and neural levels, we are able to shed light on preferential pain learning. Note that we use the term “preferential” here to indicate a different effect of the two CSs on learning, not a preference in a choice process.

Two behavioral findings should be highlighted here. First, US valence and contingency ratings indicated stronger acquisition learning from pain cues. This finding is unlikely due to a difference in affective value only as the two CS were matched for unpleasantness. Instead, this asymmetry confirms that learning depends on the inherent threat value of stimuli or events with higher threat value leading to accelerated and stronger learning.

Secondly, preferential acquisition learning of CS^+^_pain_ versus CS^+^_tone_ was more prominent in those more fearful of pain than tone stimuli. This finding further underscores the relevance of the stimulus’ threat value but also indicates that not all individuals are equally susceptible to this characteristic in their assessment. Even though there is reason to believe that an individual’s response to a potentially threatening stimulus is biologically hardwired, there still seems to be a degree of variability in the response. Given that heightened levels of fear of pain are common amongst those with chronic pain, our observation might be particularly relevant for this patient group. In fact, we recently reported that, although differential learning was generally less pronounced in chronic back pain patients than healthy participants, patients with strong pain-related anxiety and catastrophizing showed enhanced pain-related acquisition learning (Schlitt et al. 2021). Further studies are therefore needed to explore the relevance of individual threat assessment as a modulatory factor.

In contrast to the acquisition phase, the main behavioral indices showed no differences between the US during extinction learning. This suggests that, at least in healthy participants, pain-related extinction might be less dependent on the threat value of the US. We recently reported similar findings in a study in which we compared the acquisition and extinction of cues predicting the increase or decrease of pain (aversive and appetitive learning, van der Schaaf et al. 2022). Together, these data suggest that acquisition and extinction learning are two distinct processes that follow different learning rules. Interestingly, valence ratings for the CS^+^_tone_ and CS^−^ at the last extinction trial had returned to baseline level, whereas ratings of the CS^+^_pain_ were still elevated, which could be interpreted as slower extinction of pain-related associations. Whether this finding can simply be explained by stronger acquisition in the CS^+^_pain_ condition (which requires more time to reach the same level of extinction even at the same decay rate) or is indeed indicative of residual fear learning should be explored in further studies.

### Neural findings

Behavioral findings of enhanced acquisition learning were accompanied by differential activation in several key regions of the salience, fear, and extinction networks.

#### Role of the anterior insula and vmPFC

Our search for differential responses to the two CS revealed stronger activation in the left anterior insula for pain-predicting CS, which also increased over the course of the acquisition phase. The anterior insula is known to encode, integrate, and update sensory signals and emotions (Zhang et al. 2020) and is a key node of the salience network. Insular activations are affected by the threat value (Wiech et al. 2010) and salience (Menon and Uddin 2010) of a stimulus. Furthermore, the insula has been implicated in encoding sensory-specific expectancy effects (Fazeli and Büchel 2018; Sharvit et al. 2018) that are known to shape pain perception (Atlas and Wager 2012). Another potential reason for the observed insular finding might be the uncertainty regarding both US due to the 75% reinforcement schedule. Pain, for instance, might be more salient in combination with uncertainty, leading to stronger anterior insular activation as compared to the tone (for a meta-analysis see Morriss et al. 2019).

In addition to the anterior insula, the vmPFC showed stronger activity during CS^+^_pain_ than CS^+^_tone_. This is again in line with previous findings of vmPFC activation at the time of cue presentation (Sharvit et al. 2018). The vmPFC plays a key role in the representation of affective value and has recently been implicated in so-called “self-in-context models,” which represent “situations in which we find ourselves and their implications for our current and future well-being” (Koban et al. 2021). Self-in-context models focus on information that is relevant to the individual such as information on bodily integrity and well-being. Stronger weighting of information about pain is therefore in accordance with such higher-level representation.

#### Amygdala activity scales with pain-related fear during acquisition and extinction learning

The amygdala is a key brain region in fear and fear conditioning (LeDoux 1993; LaBar et al. 1998; Johnson and LeDoux 2004; Krabbe et al. 2018; Sun et al. 2020; Wen et al. 2022), and its activity has been shown to decrease across extinction (LaBar et al. 1998). However, it is rarely reported to be active during fear learning and extinction (for meta-analyses see Fullana et al. 2016, 2018), presumably because learning studies often only averaged across acquisition trials or extinction trials, ignoring dynamic aspects of learning (Morriss et al. 2018). By focusing on changes over time, we found that amygdala engagement increases more strongly for the CS^+^_pain_ compared to CS^+^_tone_ over the course of the acquisition period the more fearful participants were of painful than auditory stimulation. Of note, individual fear differences did not completely explain the preferential acquisition of pain versus tone cues, which is in line with the assumption of preferential learning for pain beyond the general effect of aversiveness. Furthermore, although extinction slopes did not differ between CS^+^_pain_ and CS^+^_tone_ and were not modulated by fear of pain, amygdala activity was. Participants who were more fearful of pain still showed increased amygdala to the CS^+^_pain_ compared to the CS^+^_tone_, even though the CS were no longer reinforced. Although speculative at this point, this residual amygdala activity might indicate resistance to cue-outcome updating despite a change in contingencies. A similar observation was previously reported by Atlas et al. 2016. Following instructed reversals of contingencies, activation changed in key regions of aversive learning except for the amygdala which continued to respond in accordance with the original reinforcement scheme. Residual amygdala activity might also be explained by individual factors other than differences in fear of pain. Morriss et al. 2015, for instance, report that, aside from trait anxiety (Barrett and Armony 2009; Sehlmeyer et al. 2011), threat uncertainty sensitivity is a potential factor associated with stronger amygdala activity during fear extinction learning in response to the CS^+^ versus CS^−^. Whether the residual amygdala activity we found is of relevance for our understanding of preferential learning of pain predicting cues or the chronification of pain remains to be investigated.

A few limitations of the study should be mentioned. First, our findings do not allow for conclusions regarding pain specificity in the strict sense. For this, one would have to compare the noxious stimulation to another stimulus identical to pain in all relevant aspects (e.g. sensory component, unpleasantness) except for the painful quality, which is notoriously difficult. Follow-up studies should nonetheless use stimuli that share characteristics with pain (e.g. itch, unpleasant odors) but also non-sensory experiences such as social pain or monetary loss to isolate properties that cause preferred pain-related learning. Second, because we only obtained valence ratings on every fourth trial we were unable to characterize rating changes with greater temporal resolution. While our ratings indicate a steady increase in negative valence for both CS^+^ over the course of the acquisition phase and a continuous decrease in the extinction phase, a more fine-grained assessment would provide further insights into the learning process. Third, larger-scale investigations are needed to further explore neural processes underlying the potentially unique function of pain-related associations in learning.

## Conclusion and outlook

Our study demonstrates that healthy participants show stronger acquisition learning for pain-predicting than tone-predicting cues, which was amplified in those with relatively higher levels of fear of pain. In line with the interpretation of this finding as indicative of the biological relevance of pain, these behavioral effects were accompanied by an engagement of brain regions implicated in threat processing (insula, amygdala) and personal significance (vmPFC). By contrast, extinction learning seems to be less dependent on the threat value of a US, at least in healthy individuals. An exception seems to be the amygdala, which showed residual activity modulated by the relatively higher fear of pain. Whether such prioritization of pain-related information can be found in patients with chronic pain, and particularly those with hypervigilance or fear of pain and how such a prioritization may contribute to the persistence of pain states remains to be investigated.

## Supplementary Material

Supplementary_Material_final_file_bhad236

## Data Availability

All data and information can be provided upon request.
