# Supplementary material for: Neural underpinnings of preferential pain learning and the modulatory role of fear
Source: Cereb Cortex. 2023 Jul 5;33(16):9664–76. doi: 10.1093/cercor/bhad236 (PMC11648315; doi:10.1093/cercor/bhad236)
Supplement: Supplementary_Material_final_file_bhad236 [file supplementary_material_final_file_bhad236.docx]

## Supplement

**Supplementary methods**

*Anatomical data preprocessing*

T1-weighted (T1w) images were corrected for intensity non-uniformity (INU) with N4BiasFieldCorrection (Tustison et al. 2010), distributed with ANTs 2.2.0 (Avants et al. 2008), and used as T1w-reference throughout the workflow. The T1w-reference was then skull-stripped using antsBrainExtraction.sh (ANTs 2.2.0), using OASIS30ANTs as target template. Spatial normalization to the ICBM 152 Nonlinear Asymmetrical template version 2009c (Fonov et al. 2009) was performed through nonlinear registration with antsRegistration (ANTs 2.2.0), using brain-extracted versions of both T1w volume and template. Brain tissue segmentation of cerebrospinal fluid (CSF), white-matter (WM) and gray-matter (GM) was performed on the brain-extracted T1w using fast (FSL 5.0.9, Zhang et al. 2001).

*Functional data preprocessing*

For each of the two sessions (acquisition, extinction), the following preprocessing was performed for each subject. First, a reference volume and its skull-stripped version were generated using a custom methodology of fMRIPrep. The BOLD reference was then co-registered to the T1w reference using flirt (FSL 5.0.9, Jenkinson and Smith 2001) with the boundary-based registration (Greve and Fischl 2009) cost-function. Co-registration was configured with nine degrees of freedom to account for distortions remaining in the BOLD reference. Head-motion parameters with respect to the BOLD reference (transformation matrices, and six corresponding rotation and translation parameters) are estimated before any spatiotemporal filtering using mcflirt (FSL 5.0.9, Jenkinson et al. 2002). BOLD runs were slice-time corrected using 3dTshift from AFNI 20160207 (Cox and Hyde 1997). The BOLD time-series (including slice-timing correction when applied) were resampled onto their original, native space by applying a single, composite transform to correct for head-motion and susceptibility distortions. These resampled BOLD time-series will be referred to as preprocessed BOLD in original space, or just preprocessed BOLD. The BOLD time-series were resampled to MNI152NLin2009cAsym standard space, generating a preprocessed BOLD run in MNI152NLin2009cAsym space. First, a reference volume and its skull-stripped version were generated using a custom methodology of fMRIPrep. Several confounding time-series were calculated based on the preprocessed BOLD: framewise displacement (FD), DVARS and three region-wise global signals. FD and DVARS are calculated for each functional run, both using their implementations in Nipype (following the definitions by Power et al. 2014). The three global signals are extracted within the CSF, the WM, and the whole-brain masks. Additionally, a set of physiological regressors were extracted to allow for component-based noise correction (CompCor, Behzadi et al. 2007). Principal components are estimated after high-pass filtering the preprocessed BOLD time-series (using a discrete cosine filter with 128s cut-off) for the two CompCor variants: temporal (tCompCor) and anatomical (aCompCor). Six tCompCor components are then calculated from the top 5% variable voxels within a mask covering the subcortical regions. This subcortical mask is obtained by heavily eroding the brain mask, which ensures it does not include cortical GM regions. For aCompCor, six components are calculated within the intersection of the aforementioned mask and the union of CSF and WM masks calculated in T1w space, after their projection to the native space of each functional run (using the inverse BOLD-to-T1w transformation). The head-motion estimates calculated in the correction step were also placed within the corresponding confounds file. All resamplings can be performed with a single interpolation step by composing all the pertinent transformations (i.e. head-motion transform matrices, susceptibility distortion correction when available, and co-registrations to anatomical and template spaces). Gridded (volumetric) resamplings were performed using antsApplyTransforms (ANTs), configured with Lanczos interpolation to minimize the smoothing effects of other kernels (Lanczos 1964). Non-gridded (surface) resamplings were performed using mri_vol2surf (FreeSurfer).

*Additional References*

Avants B, Epstein C, Grossman M, Gee J. 2008. Symmetric diffeomorphic image registration with cross-correlation: Evaluating automated labeling of elderly and neurodegenerative brain. Medical Image Analysis. 12:26–41.

Fonov V, Evans A, McKinstry R, Almli C, Collins D. 2009. Unbiased nonlinear average age-appropriate brain templates from birth to adulthood. NeuroImage. 47:S102.

Greve DN, Fischl B. 2009. Accurate and robust brain image alignment using boundary-based registration. NeuroImage. 48:63–72.

Power JD, Mitra A, Laumann TO, Snyder AZ, Schlaggar BL, Petersen SE. 2014. Methods to detect, characterize, and remove motion artifact in resting state fMRI. NeuroImage. 84:320–341.

Tustison NJ, Avants BB, Cook PA, Yuanjie Zheng, Egan A, Yushkevich PA, Gee JC. 2010. N4ITK: Improved N3 Bias Correction. IEEE Trans Med Imaging. 29:1310–1320.

Zhang Y, Brady M, Smith S. 2001. Segmentation of brain MR images through a hidden Markov random field model and the expectation-maximization algorithm. IEEE Trans Med Imaging. 20:45–57.

# Supplementary data

**Figures and Tables**


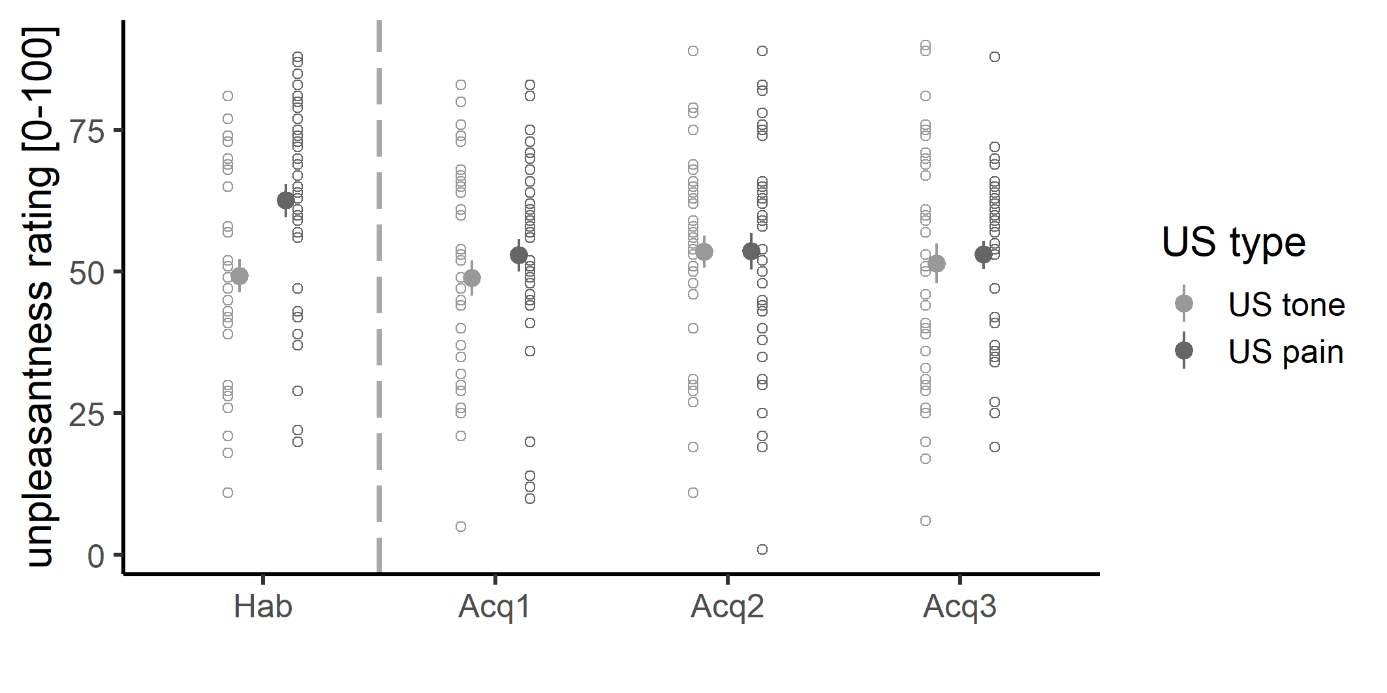


***Figure S1*:** Unpleasantness ratings provided during habituation (Hab) and acquisition training (Acq1 – Acq3) for the US pain (dark gray) and US tone (light gray). Displayed are means (filled circles) and single subject data (empty circles). Error bars indicate standard error of the mean.


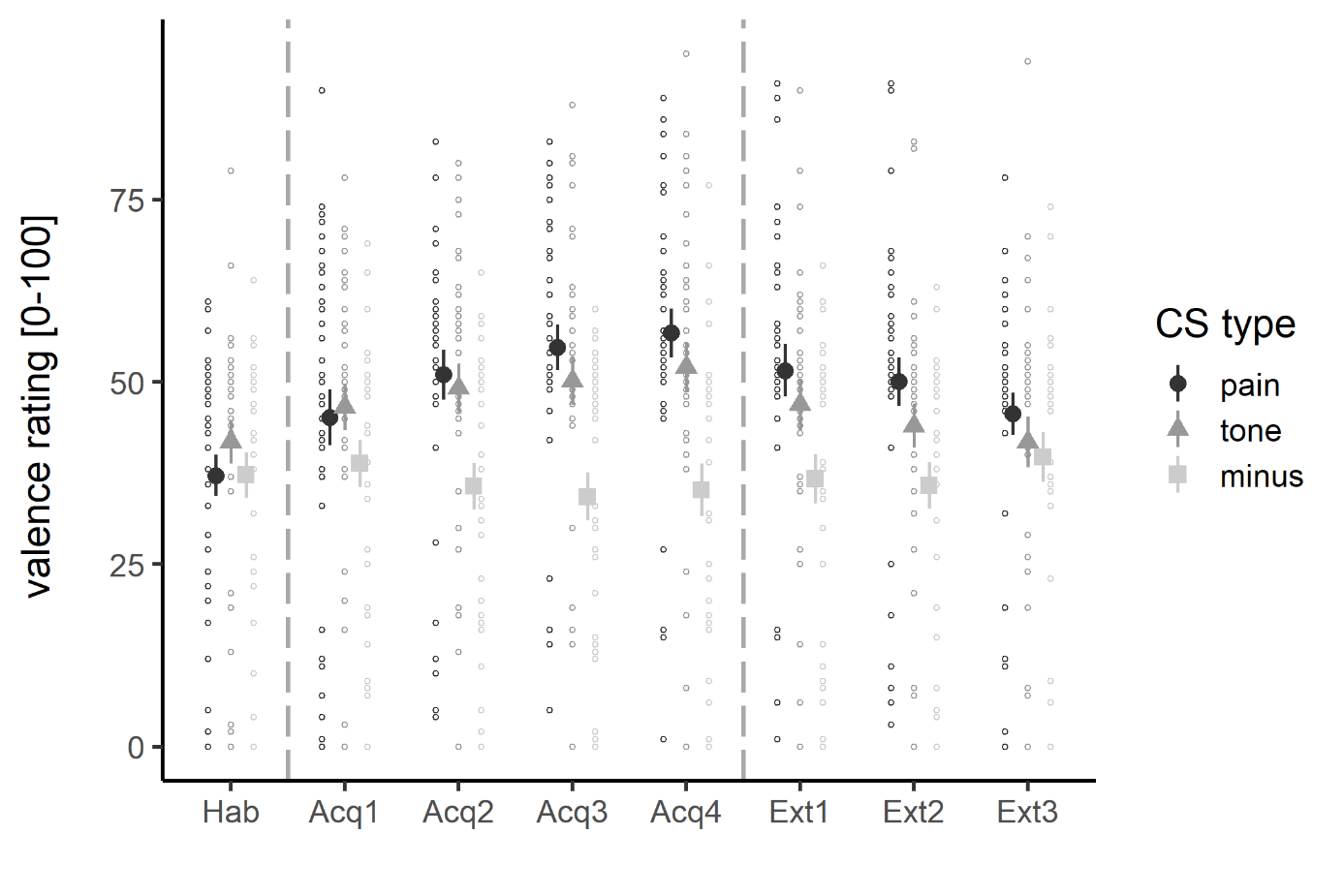

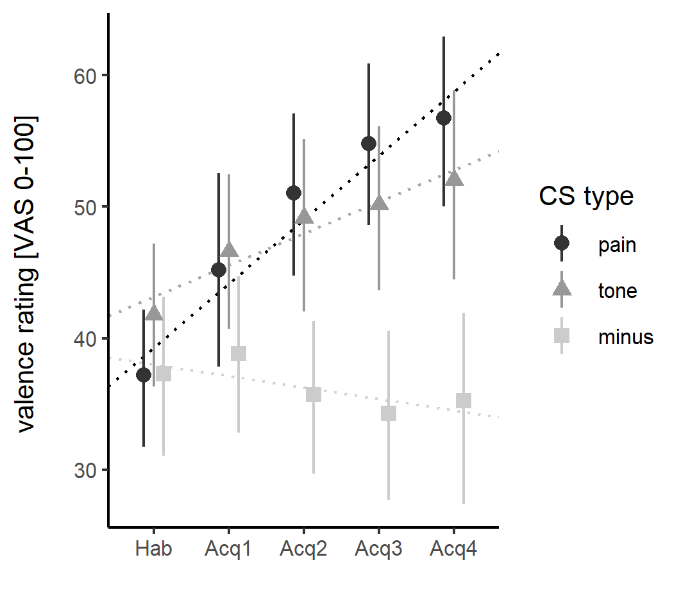

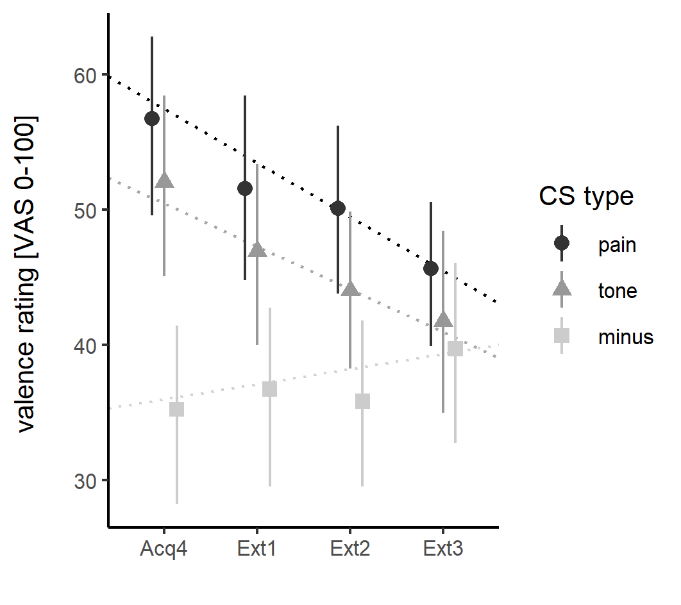


**(B)**

**(A)**

**Figure S2:** (A) CS valence ratings [VAS 0-100] provided during the three experimental phases of the differential conditioning paradigm for each CS types CS^-^ (rectangle), CS^+^_pain_ (circle) or CS^+^_tone_ (triangle): habituation phase (Hab), acquisition training (Acq1- Acq4) and extinction training (Ext1 – Ext3). Displayed are means and single data. Error bars indicate standard error of the mean. Values above the horizontal dashed line indicate negative valence while values below indicate positive valence. VAS anchors were labelled as 0 = “very pleasant”, 50 = “neutral”, 100 = “very unpleasant”. (B) For visualization purposes regression lines are overlaid on the CS valence ratings [VAS 0-100] provided during the three experimental phases of the differential conditioning paradigm as given by the two linear mixed models for the acquisition phase (left) and the extinction phase (right).


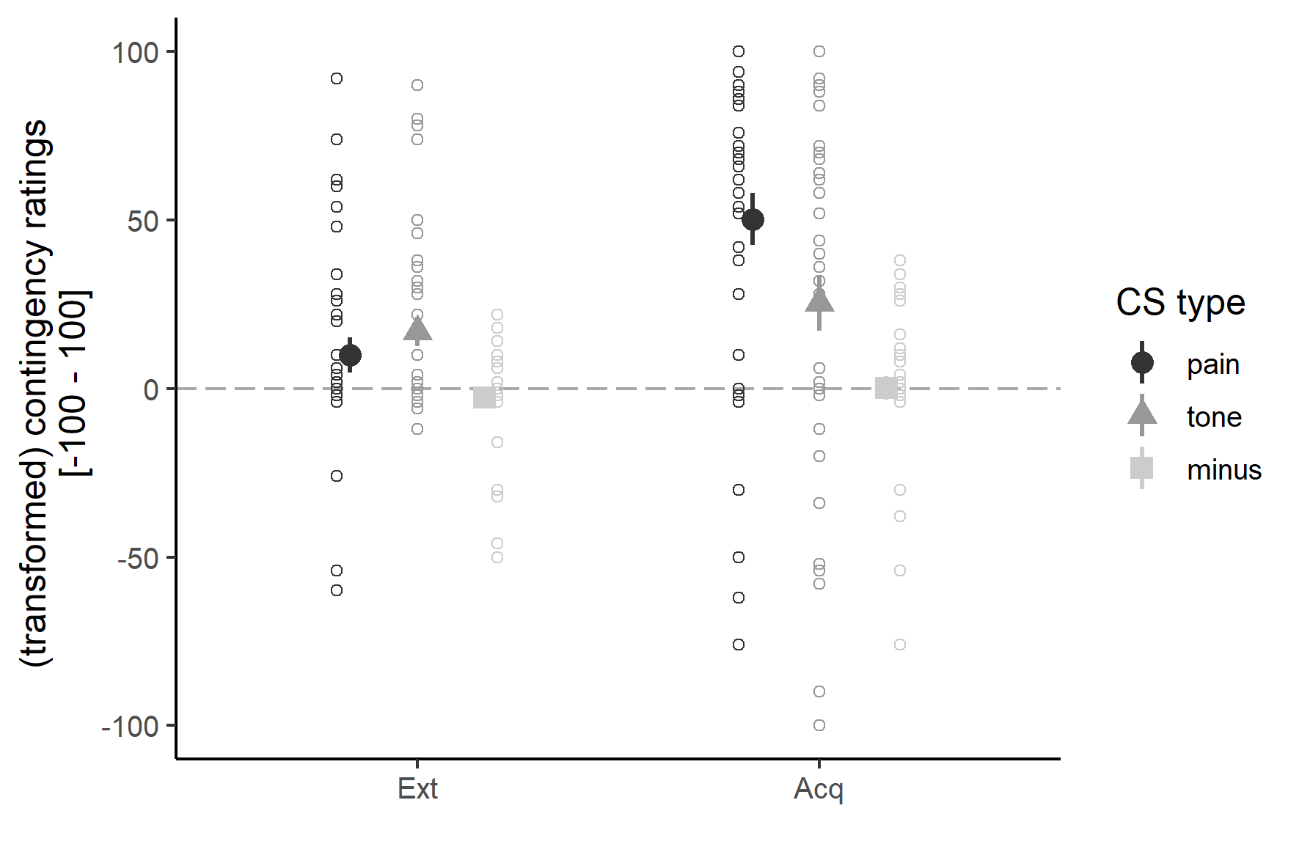


***Figure S3****:* US-CS contingency ratings as provided after acquisition training (Acq) and extinction training (Ext). Depicted are means (filled circles) standard error of the mean and single participant data (empty circles) for each CS type separately (rectangle = CS^-^, triangle = CS^+^ _tone_, circle = CS^+^ _pain_. Anchors VAS contingency: -100 = “100% pain”, 50 = “0% stimulation, 100 = “100% tone”. Raw US-CS contingency ratings were transformed (see methods) to range from -100 to 100. As a result, negative values indicate wrong US-CS assignments (e.g. CS^+^ _pain_ was incorrectly assigned to the tone).

***
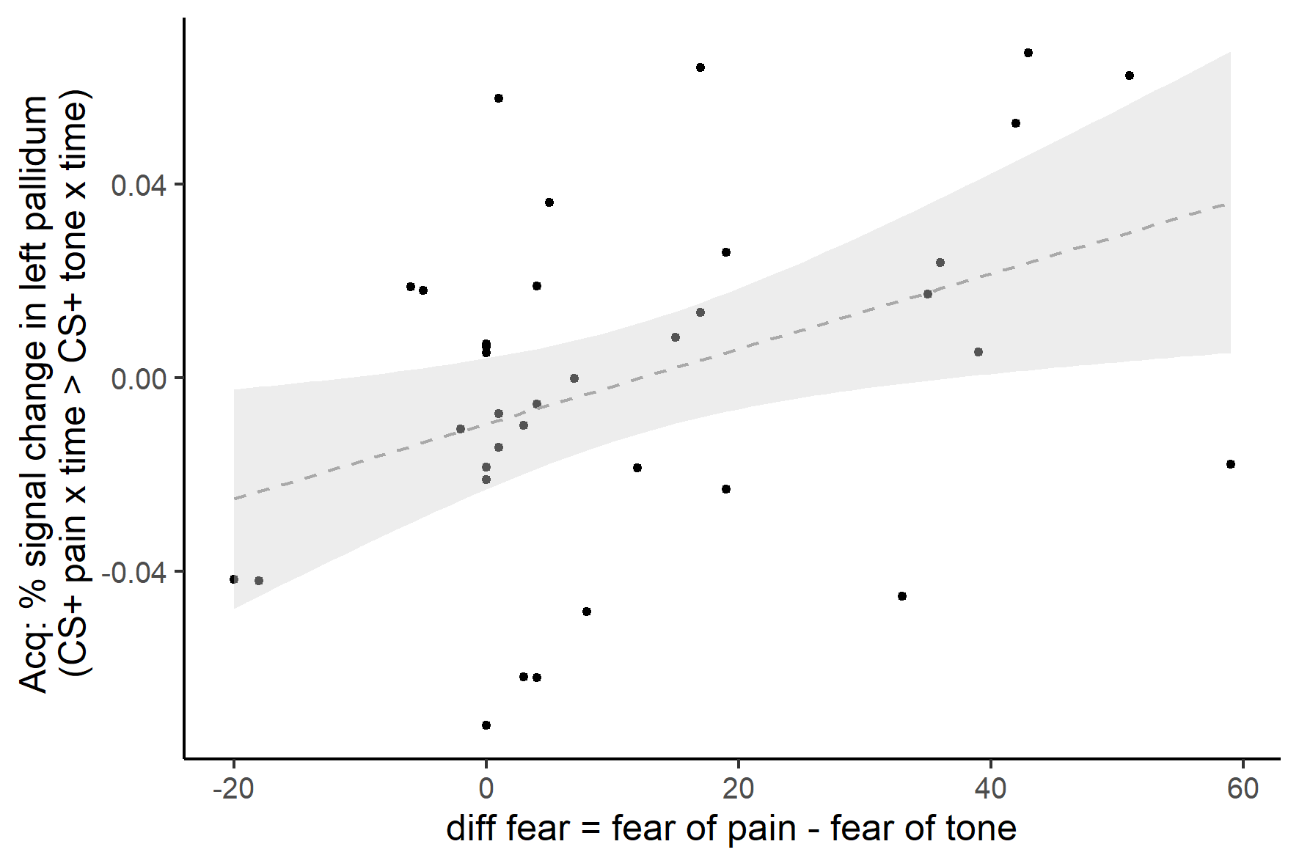
***

***Figure S4****:* Pain-specific activation changes in the left pallidum were associated with individual differences in fear ratings (see also Figure 5 and Table 2). For visualization purposes % signal change in the left pallidum (peak voxel + 5 mm sphere) is plotted against individual differences in fear ratings (diff fear). Black circles depict single participant data. The solid line and gray area represent the regression line and 95% confidence interval, respectively.


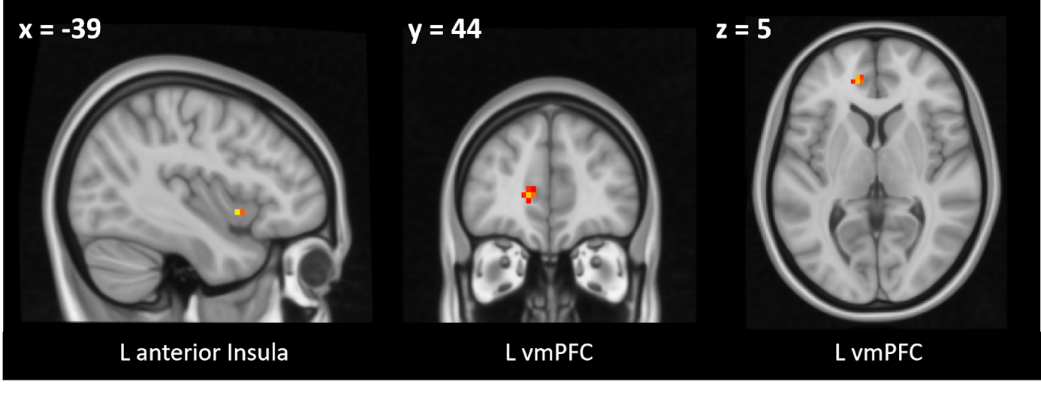
 **(A)**


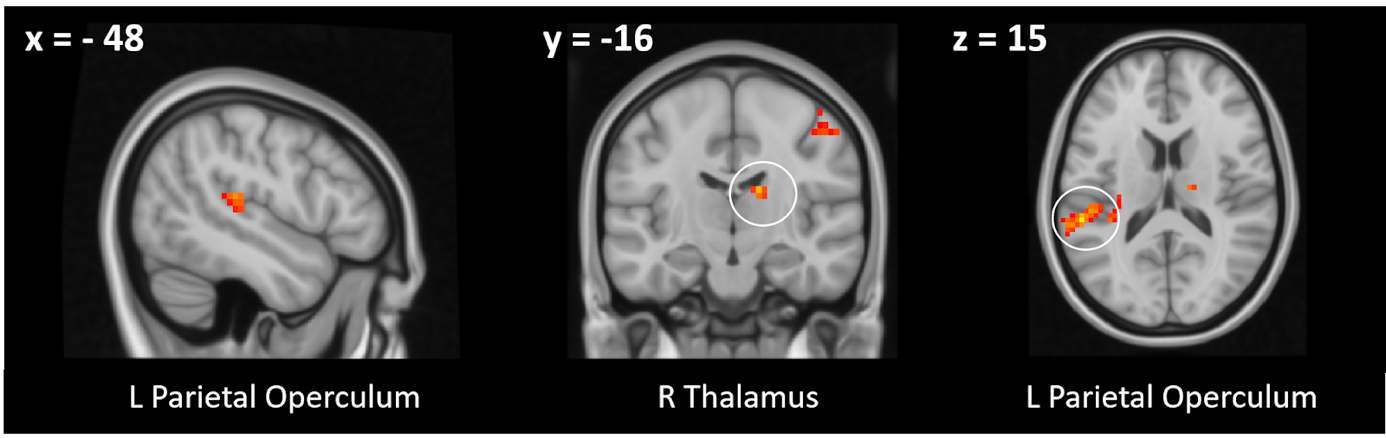
 **(B)**

**Figure S5:** Modality-specific neural responses to cues (CS^+^) predicting pain versus aversive tone during acquisition training. (A) CS^+^_pain_ > CS^+^_tone_  and (B) CS^+^_tone_  > CS^+^_pain_ . Significant neural activations are superimposed on a structural T1-image and thresholded at p < 0.001, k > 0, uncorrected for visualization purposes.

**Figure S6:** Increasing amygdala activation for pain-predicting vs. tone-predicting cues during acquisition training scaled with stronger fear of pain than fear of tone. For visualization purposes, Figure (A) – (D) show an overlay of the observed amygdala activation (in shades of red; thresholded at p < 0.001, k = 0) onto (A) a mask of the right amygdala as defined in the Harvard-Oxford cortical and subcortical structural atlases (in yellow) and (B-D) an activation map (association test map; in blue) based on an automated meta-analysis of 21 studies included in the neurosynth database using the search term “fear” (<https://neurosynth.org/analyses/terms/fear/>).


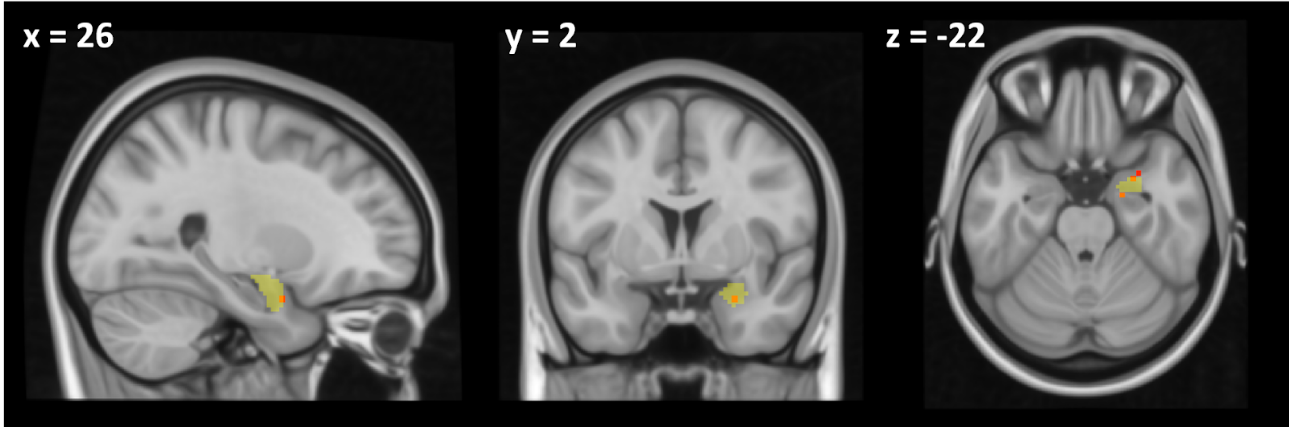

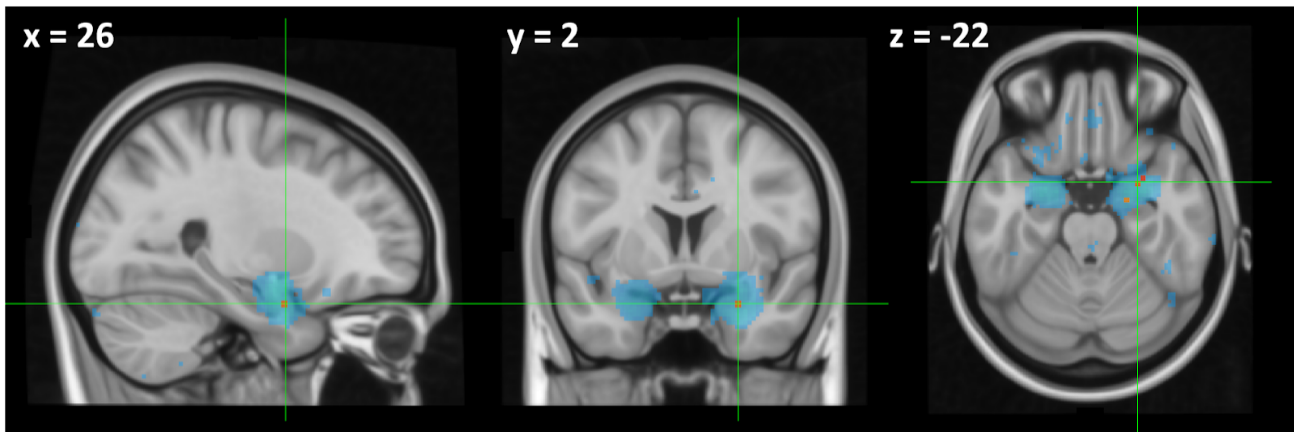

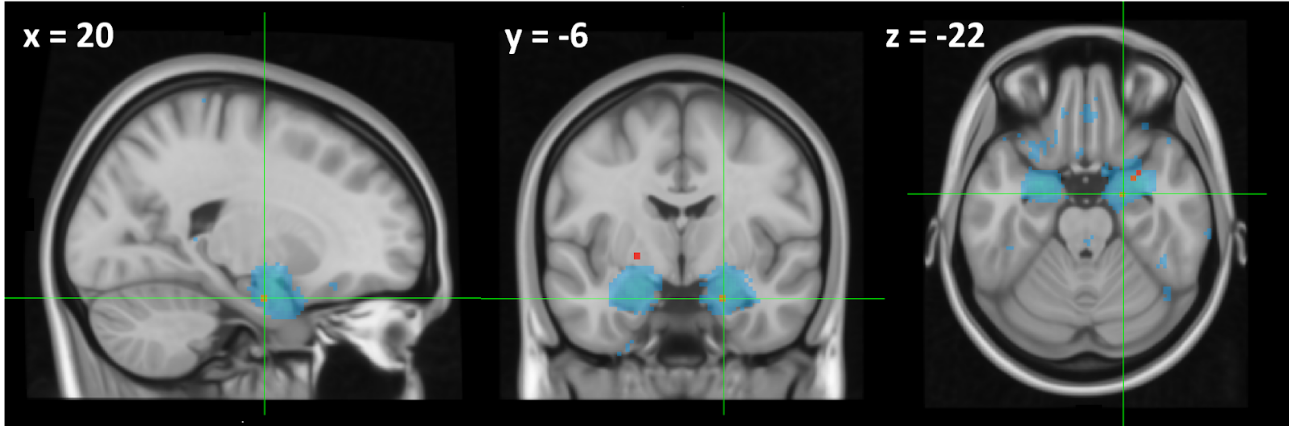

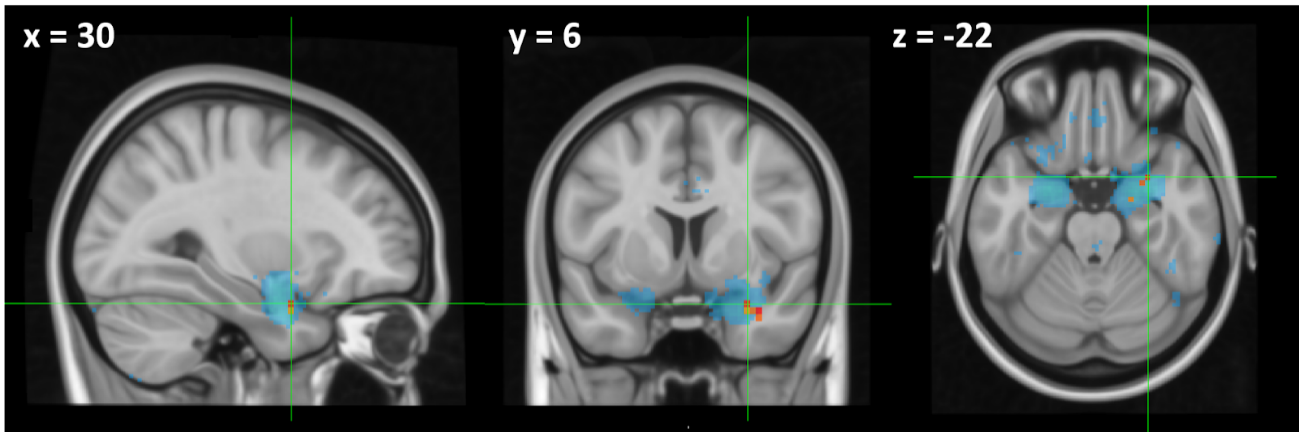


**(A)**

**(B)**

**(C)**

**(D)**

**Figure S7:** Enhanced activity within the right amygdala for pain-predicting than tone-predicting cues during extinction training for participants reporting higher fear of painful vs. auditory stimulation. For visualization purposes, Figure (A) – (D) show an overlay of the observed amygdala activation (in shades of yellow/red; thresholded at p < 0.001, k = 0) onto a (A) - (B) mask of the right amygdala as defined in the Harvard-Oxford cortical and subcortical structural atlases (in turquoise) and (C) - (D) an activation map (association test map; in blue) based on an automated meta-analysis of 21 studies included in the neurosynth database using the search term “fear” (<https://neurosynth.org/analyses/terms/fear/>).


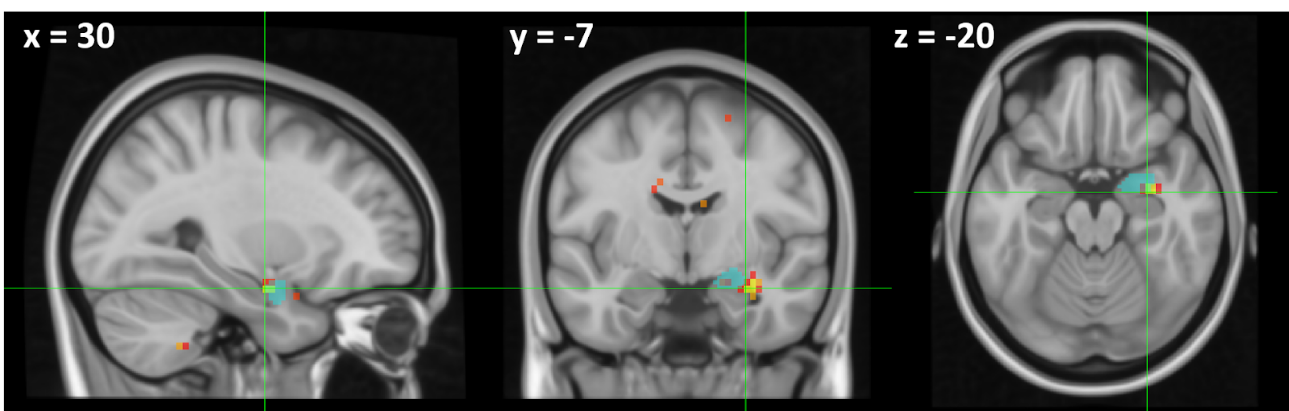

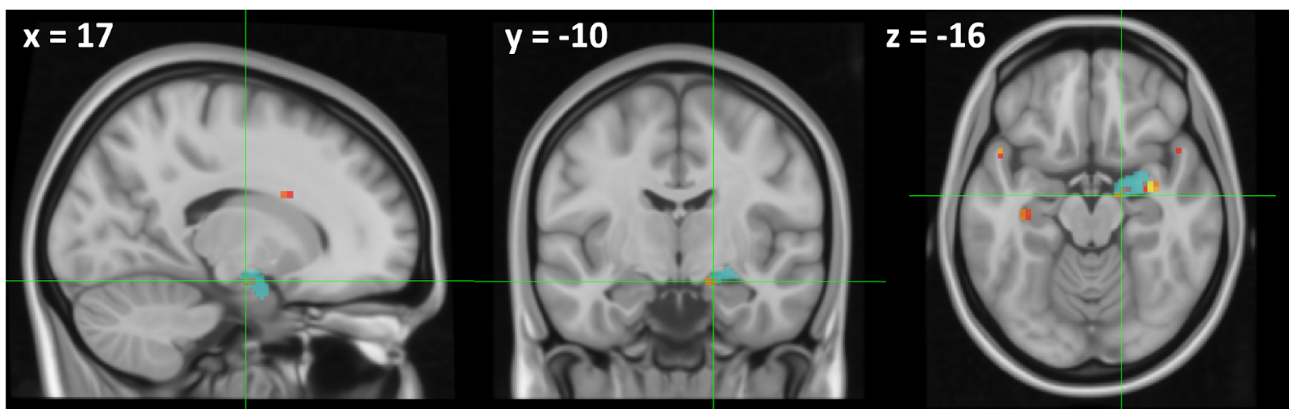

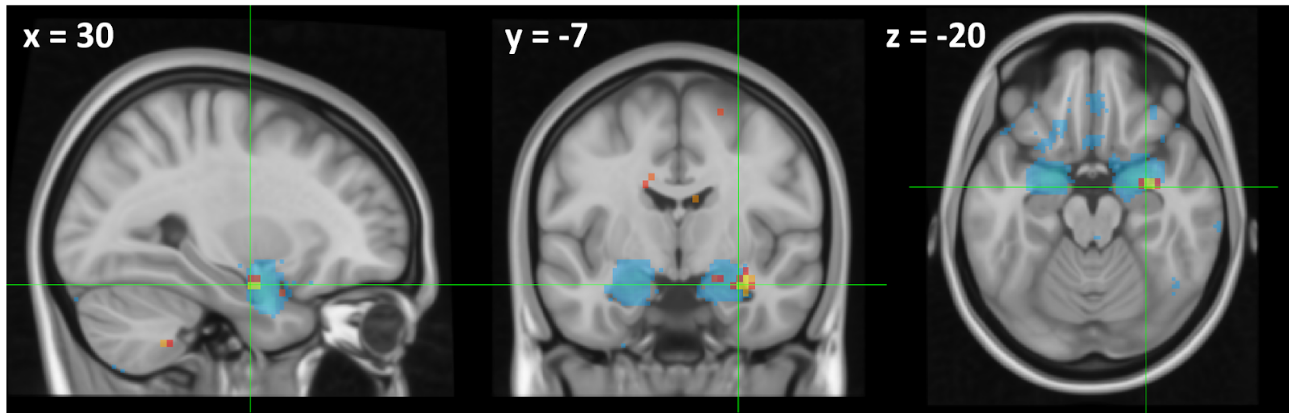

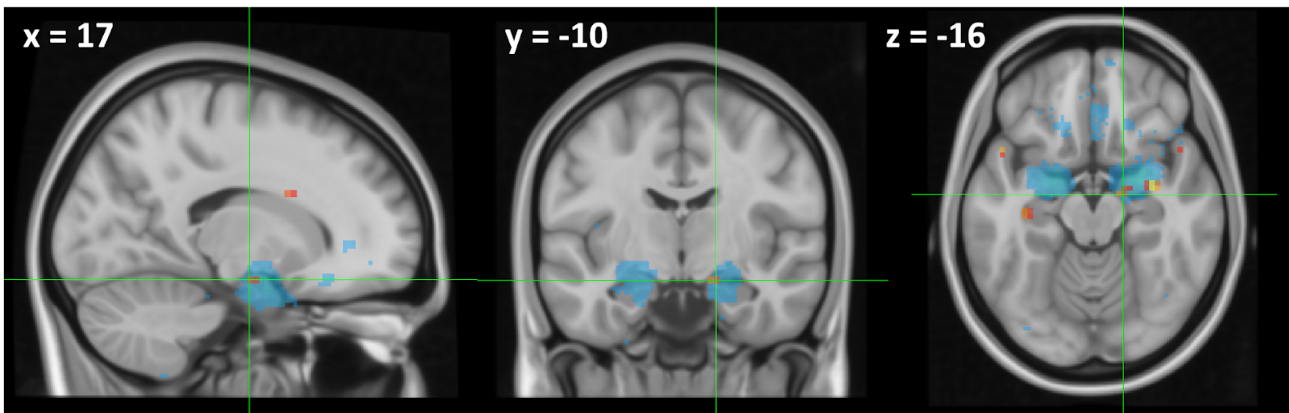


**(A)**

**(B)**

**(C)**

**(D)**

**Table S1:** Questionnaire data of the study sample (N = 38).

| **Questionnaire** | **M** | **SD** |
| --- | --- | --- |
| *CES-D* | 7.03 | 7.05 |
| *PCS* | 16.89 | 9.28 |
| *PASS D1* | 2.24 | 1.08 |
| *PASS D2* | 1.66 | 0.78 |
| *PASS D3* | 1.16 | 0.91 |
| *PASS D4* | 1.01 | 0.76 |

*Abbreviations*: CES-D, Center for Epidemiologic Studies – Depression Scale; PCS, Pain Catastrophizing Scale; PASS D1, Pain Anxiety Symptoms Scale, subscale Cognitive; PASS D2, subscale Escape/avoidance; PASS D3, subscale Fear; PASS-D4 subscale Physiological anxiety

**Acquisition**

***Table S2:*** US-specific BOLD-responses during acquisition training; FWE whole brain corrected, p < 0.05.

|  |  | **MNI-coordinates** | | | |  |  |  |
| --- | --- | --- | --- | --- | --- | --- | --- | --- |
| **Contrast** | **Region** | **H** | **x** | **y** | **z** | **T** | **k** | **P** |
| **US pain > US tone** | | | | | | | | |
|  | Posterior insula | R | 41 | -4 | 8 | 10.98 | 285 | < 0.001* |
|  | Posterior insula | L | -36 | -19 | 15 | 6.98 | 3 | 0.001* |
|  | Central opercular Cortex | L | -36 | 2 | 12 | 9.38 | 57 | < 0.001* |
|  | Superior parietal lobe | R | 20 | -45 | 67 | 9.33 | 22 | < 0.001* |
|  | Precentral gyrus | L | -57 | 5 | 8 | 7.7 | 21 | 0.001* |
|  | Supramarginal Gyrus, anterior | L | -63 | -25 | 29 | 7.09 | 20 | 0.001* |
|  | Paracingulate Gyrus | R | 6 | 14 | 43 | 6.6 | 3 | 0.003* |
| **US tone > US pain** | | | | | | | | |
|  | Planum Temporale | L | -39 | -30 | 12 | 11.95 | 399 | < 0.001* |
|  | Superior Temporal Gyrus, posterior | R | 62 | -27 | 8 | 10.66 | 382 | < 0.001* |
|  | Postcentral Gyrus | R | 53 | -10 | 46 | 5.73 | 1 | 0.044* |

Differential neural activation induced by painful stimulation (US pain) versus auditory stimulation (US tone). Exact p-values and coordinates are shown for the peak voxel of whole brain analysis (P_FWE_ < 0.05, *). Abbreviations: H, hemisphere; k, cluster size; L, left; R, right; US, unconditioned stimulus.

***Table S3:*** Modality-related neural responses to cues (CS^+^) predicting pain (CS^+^_pain_ > CS^-^) or an aversive tone (CS^+^_tone_ > CS^-^) during acquisition training and their modulation by modality-specific fear ratings.

|  |  | **MNI-coordinates** | | | |  |  |
| --- | --- | --- | --- | --- | --- | --- | --- |
| **Contrast** | **Region** | **H** | **x** | **y** | **z** | **T** | **P** |
| *Differential contrasts* | | | | | | | |
| **CS^+^_pain_ > CS^-^** | | | | | | | |
|  | Anterior insula | R | 38 | 11 | -6 | 3.82 | 0.035* |
|  | SII/Parietal operculum | R | 53 | -22 | 19 | 3.31 | 0.04* |
| **CS^-^ > CS^+^_pain_** | | | | | | | |
|  | Thalamus | R | 3 | -10 | 5 | 3.94 | 0.047* |
|  | Cerebellum | R | 35 | -60 | -43 | 4.92 | 0.025* |
|  | *Middle frontal gyrus* | *L* | *-33* | *32* | *22* | *4.63* | *< 0.001* |
| **CS^+^_tone_ > CS^-^** | | | | | | | |
|  | SII/Parietal operculum | R | 50 | -22 | 15 | 4.33 | 0.003* |
|  | Heschl gyrus | L | -42 | -25 | 12 | 2.91 | 0.037* |
|  | Heschl gyrus | R | 50 | -16 | 8 | 4.00 | 0.002* |
|  | *Planum temporale/*  *primary auditory cortex* | *L* | *-48* | *-30* | *8* | *4.58* | *< 0.001* |
|  | *STG, posterior* | *L* | *-57* | *-36* | *12* | *4.17* | *< 0.001* |
| **CS^-^ > CS^+^_tone_** | | | | | | | |
|  | *Cerebellum* | *R* | *35* | *-81* | *-33* | *4.05* | *< 0.001* |
| *Modulation by individual fear ratings* | | | | | | | |
| **CS^+^_pain_ > CS^-^ x fear of pain** | | | | | | | |
|  | *Cerebellum* | *R* | *6* | *-60* | *-19* | *4.68* | *< 0.001* |
| **CS^+^_pain_ < CS^-^ x fear of pain** | | | | | | | |
|  | - | - | - | - | - | - | - |
| **CS^+^_tone_ > CS^-^ x fear of tone** | | | | | | | |
|  | Parahippocampal gyrus, posterior | L | -15 | -36 | -12 | 3.91 | 0.005* |

Modality-related neural responses to cues predicting pain (CS^+^_pain_) or an aversive tone (CS^+^_tone_) as compared to a cue signaling no subsequent stimulation (CS^-^) during acquisition training and their modulation by modality-specific fear ratings. Peak voxel indicate significant activation after small volume correction using pre-defined ROIs (P_FWE_ < 0.05, *) or uncorrected whole-brain analyses (in italic font, cluster size k ≥ 10; all p < 0.001), respectively. Exact unilateral p-values are provided.

Abbreviations: CS, conditioned stimulus; H, hemisphere; L, left; R, right

***Table S4:*** Time x condition interaction: Increasing modality-related neural responses to cues (CS^+^) predicting pain (CS^+^_pain_ > CS^-^) or tone (CS^+^_tone_ > CS^-^) during acquisition training and their modulation by modality-specific fear ratings.

|  |  | **MNI-coordinates** | | | |  |  |
| --- | --- | --- | --- | --- | --- | --- | --- |
| **Contrast** | **Region** | **H** | **x** | **y** | **z** | **T** | **P** |
| *Time by condition interaction* | | | | | | | |
| **CS^+^_pain x time_ > CS^-^ _time_** | | | | | | | |
|  | - | - | - | - | - | - | - |
| **CS^-^ _time_ > CS^+^ _pain x time_** | | | | | | | |
|  | - | - | - | - | - | - | - |
| **CS^+^_tone x time_ > CS^-^ _time_** | | | | | | | |
|  | - | - | - | - | - | - | - |
| **CS^-^ _time_ > CS^+^_tone x time_** | | | | | | | |
|  | - | - | - | - | - | - | - |
| *Modulation by individual fear* | | | | | | | |
| **CS^+^_pain_ _x time_ > CS^-^ _time_ x fear of pain** | | | | | | | |
|  | Amygdala | R | 29 | -1 | -26 | 4.06 | 0.01* |
| **CS^+^_pain_ _x time_ < CS^-^ _time_ x fear of pain** | | | | | | | |
|  | Hippocampus | R | 32 | -19 | -19 | 3.75 | 0.04* |
|  | Pallidum | R | 17 | -7 | -6 | 3.97 | 0.011* |
|  | *Inferior Temporal gyrus* | *R* | *50* | *-51* | *-6* | *4.91* | *< 0.001* |
| **CS^+^_tone_ _x time_ > CS^-^ _time_ x fear of tone** | | | | | | | |
|  | Caudate nucleus | L | -9 | 20 | 1 | 4.42 | 0.005* |
|  | *Inferior frontal gyrus* | *L* | *-51* | *32* | *-2* | *3.83* | *< 0.001* |
| **CS^+^_tone_ _x time_ < CS^-^ _time_ x fear of tone** | | | | | | | |
|  | Pallidum | R | 23 | -10 | -2 | 5.36 | < 0.001 |
|  | Pallidum | R | 17 | -4 | 5 | 3.94 | 0.012* |

Time- and modality-related neural responses to cues predicting pain (CS^+^_pain_) or tone (CS^+^_tone_) as compared to a cue signaling no subsequent stimulation (CS^-^) during acquisition training and their modulation by modality-specific fear ratings. Peak voxel indicate significant activation after small volume correction using pre-defined ROIs (P_FWE_ < 0.05, *) or uncorrected whole-brain analyses (in italic font, cluster size k ≥ 10; all p < 0.001), respectively. Exact unilateral p-values are provided.

Abbreviations: CS, conditioned stimulus; H, hemisphere; L, left; R, right

**Extinction**

***Table S5:*** Modality-related neural responses to cues (CS^+^) predicting pain (CS^+^_pain_ > CS^-^) or an aversive tone (CS^+^_tone_ > CS^-^) during extinction training and their modulation by modality-specific fear ratings.

|  |  | **MNI-coordinates** | | | |  |  |
| --- | --- | --- | --- | --- | --- | --- | --- |
| **Contrast** | **Region** | **H** | **x** | **y** | **z** | **T** | **P** |
| *Differential contrast* | | | | | | | |
| **CS^+^_pain_ > CS^-^** | | | | | | | |
|  | - | - | - | - | - | - | - |
| **CS^-^ > CS^+^_pain_** | | | | | | | |
|  | *Cuneal cortex* | *R* | *14* | *-78* | *29* | *4.68* | *< 0.001* |
|  | *Lingual gyrus* | *L* | *-15* | *-48* | *-9* | *4.53* | *< 0.001* |
|  | *Postcentral gyrus* | *R* | *9* | *-45* | *67* | *3.87* | *< 0.001* |
| **CS^+^_tone_ > CS^-^** | | | | | | | |
|  | - | - | - | - | - | - | - |
| **CS^-^ > CS^+^_tone_** | | | | | | | |
|  | Hippocampus | L | -21 | -19 | -23 | 3.7 | 0.045* |
| *Modulation by fear* | | | | | | | |
| **CS^+^_pain_ > CS^-^ x fear of pain** | | | | | | | |
|  | - | - | - | - | - | - | - |
| **CS^+^_pain_ < CS^-^ x fear of pain** | | | | | | | |
|  | *Frontal pole* | *L* | *-27* | *53* | *12* | *4.45* | *< 0.001* |
| **CS^+^_tone_ > CS^-^ x fear of tone** | | | | | | | |
|  | - | - | - | - | - | - | - |
| **CS^+^_tone_ < CS^-^ x fear of tone** | | | | | | | |
|  | - | - | - | - | - | - | - |

Modality-related neural responses to cues previously predicting pain (CS^+^_pain_) or an aversive tone (CS^+^_tone_) as compared to a cue signaling no subsequent stimulation (CS^-^) during extinction training and their modulation by modality-specific fear ratings. Peak voxel indicate significant activation after small volume correction using pre-defined ROIs (P_FWE_ < 0.05, *) or uncorrected whole-brain analyses (in italic font, cluster size k ≥ 10; all p < 0.001), respectively. Exact unilateral p-values are provided.

Abbreviations: CS, conditioned stimulus; H, hemisphere; L, left; R, right

***Table S6:*** Extinction training: Changes in modality-related neural responses to cues (CS^+^) that have previously predicted pain (CS^+^_pain_ > CS^-^) or tone (CS^+^_tone_ > CS^-^) and their modulation by modality-specific fear ratings.

|  |  | **MNI-coordinates** | | | |  |  |
| --- | --- | --- | --- | --- | --- | --- | --- |
| **Contrast** | **Region** | **H** | **x** | **y** | **z** | **T** | **P** |
| *Time by condition interaction* | | | | | | | |
| **CS^+^_pain x time_ > CS^-^ _time_** | | | | | | | |
|  | *-* | *-* | *-* | *-* | *-* | *-* | *-* |
| **CS^-^ _time_ > CS^+^_pain x time_** | | | | | | | |
|  | *Inferior temporal gyrus* | *L* | *-51* | *-13* | *-26* | *4.32* | *< 0.001* |
| **CS^+^_tone x time_ > CS^-^ _time_** | | | | | | | |
|  | Cingulate gyrus, posterior | R | 6 | -42 | 32 | 3.90 | 0.03* |
| **CS^-^ _time_ > CS^+^_tone x time_** | | | | | | | |
|  | *Supramarginal gyrus, posterior* | *L* | *-42* | *-51* | *53* | *4.52* | *< 0.001* |
|  | *Lingual gyrus* | *L* | *-21* | *-63* | *1* | *4.45* | *< 0.001* |
|  | *Precentral gyrus* | *R* | *29* | *-4* | *46* | *4.45* | *< 0.001* |
|  | *Cuneal cortex/visual cortex V1* | *L* | *-12* | *-81* | *15* | *4.34* | *< 0.001* |
|  | *Intracalcarine Cortex* | *L* | *-9* | *-69* | *12* | *3.72* | *< 0.001* |
| *Modulation by individual fear* | | | | | | | |
| **CS^+^_pain x time_ > CS^-^ _time_ x fear ratings pain** | | | | | | | |
|  | *Precentral gyrus* | *L* | *-27* | *-13* | *53* | *4.12* | *< 0.001* |
| **CS^+^_pain x time_ < CS^-^ _time_ x fear ratings pain** | | | | | | | |
|  | Thalamus | L | -12 | -10 | 5 | 4.35 | 0.016* |
| **CS^+^_tone x time_ > CS^-^ _time_ x fear ratings tone** | | | | | | | |
|  | - | - | - | - | - | - | - |
| **CS^+^_tone x time_ < CS^-^ _time_ x fear ratings tone** | | | | | | | |
|  | Amygdala | R | 26 | -7 | -16 | 3.86 | 0.018* |

Time- and modality-related neural responses to cues previously predicting pain (CS^+^_pain_) or tone (CS^+^_tone_) as compared to a cue signaling no subsequent stimulation (CS^-^) during extinction training and their modulation by modality-specific fear ratings. Peak voxel indicate significant activation after small volume correction using pre-defined ROIs (P_FWE_ < 0.05, *) or uncorrected whole-brain analyses (in italic font, cluster size k ≥ 10; all p < 0.001), respectively. Exact unilateral p-values are provided.

Abbreviations: CS, conditioned stimulus; H, hemisphere; L, left; R, right
